# Four Steroidal Saponins Isolated from the Aerial Parts of *Allium jesdianum* Exhibit Antibiofilm Effects Against Colistin-Resistant Clinical Strains, with an In Silico Study

**DOI:** 10.5812/ijpr-169955

**Published:** 2026-05-04

**Authors:** Saeid Eslami, Behzad Zolfaghari, Masoud Sadeghi Dinani, Arezoo Mirzaee, Hajar Sirous, Mustafa Ghanadian

**Affiliations:** 1 Department of Pharmacognosy, School of Pharmacy, Isfahan University of Medical Sciences, Isfahan, Iran; 2 Department of Bacteriology and Virology, Faculty of Medicine, Isfahan University of Medical Science, Isfahan, Iran; 3 Department of Bioinformatics, Bioinformatics Research Center, School of Pharmacy and Pharmaceutical Sciences, Isfahan University of Medical Sciences, Isfahan, Iran; 4 Department of Pharmacognosy, Isfahan University of Medical Sciences, Isfahan, Iran

**Keywords:** *Allium jesdianum*, Steroidal Saponins, Efflux Pumps, Multidrug resistance, Colistin-resistant, Anti-biofilm, Molecular docking

## Abstract

**Background:**

Global health security is critically threatened by multidrug-resistant Gram-negative bacteria, which frequently overexpress efflux pumps to expel antibiotics, rendering last-resort drugs like colistin ineffective and diminishing treatment options.

**Methods:**

This study investigated the antibacterial and antibiofilm potential of four steroidal saponins isolated from the aerial parts of *Allium jesdianum* Boiss. & Buhse against colistin-resistant strains of *Klebsiella pneumoniae*, *Escherichia coli*, *Acinetobacter baumannii*, and *Pseudomonas aeruginosa*. Compounds were extracted, purified using modern chromatographic techniques, and structurally characterized via NMR and mass spectrometry as known saponins, including aginoside and F-gitonin derivatives.

**Results:**

Four steroidal-type saponins were isolated. Bioactivity assays revealed that compounds 1 - 3 exhibited minimum inhibitory concentrations (MICs) of 4 mg/mL against colistin-resistant pathogens: *Klebsiella pneumoniae*, *Escherichia coli*, and *Acinetobacter baumannii*, though no bactericidal activity was observed via agar diffusion. Notably, compound 3 demonstrated the strongest antibiofilm activity, achieving up to 75% inhibition at 4 mg/mL. Quantitative real-time PCR analysis showed that all compounds upregulated efflux pump genes (adeB, acrA, blaKPC, oprL), which limit their antibacterial activity. Molecular docking studies further supported the interaction of compound 3 with the adeB efflux pump, indicating strong binding affinity via hydrogen bonding and hydrophobic interactions.

**Conclusions:**

These findings highlight the saponin profile in aerial parts of edible plant *A. jesdianum* as well as their modest antibacterial activity, but with potential as adjuvants in combating biofilm formation.

## 1. Introduction

Global health security relies on prevention, detection, and rapid response through international collaboration. The WHO coordinates efforts and research, with its previous priority pathogen list guiding preparedness against threats like *Klebsiella pneumoniae*, *Escherichia coli*, and *Acinetobacter baumannii* (1). These pathogens employ efflux pump–mediated resistance, actively expelling antimicrobial agents from their cells. This mechanism reduces intracellular drug concentrations, undermines the efficacy of existing therapies, and poses significant challenges for the development of new antimicrobials (2).

colistin, an older antibiotic regarded as a ‘last-resort’ agent, has long been used to treat Gram-negative infections resistant to multiple drugs (3). Resistance in *Klebsiella pneumoniae* is escalating, commonly driven by chromosomal alterations and plasmid-mediated mcr determinants (4). In *E. coli*, the emergence of mcr-1 genes has become a growing global concern, largely attributed to the horizontal dissemination of these genes (5). Pathogens such as *Pseudomonas aeruginosa* and *A. baumannii* commonly evade its action through lipid A modification and the activation of efflux pumps (6, 7). Various natural compounds,particularly plant-derived saponins, have effects on resistant pathogens (8).

Saponins represent a varied group of plant-derived natural compounds recognized for their therapeutic potential. They are generally divided into two categories: triterpenoid saponins, characterized by a 30-carbon backbone, and steroidal saponins, distinguished by a 27-carbon backbone (8-10). Steroidal saponins exhibit diverse biological functions, such as lowering uric acid levels, alleviating ulcers, and exerting antispasmodic effects. They also provide protective action on vital organs, including the lungs, liver, kidneys, and brain, and contribute to the prevention of disorders like atherosclerosis, arthritis, obesity, diabetes, osteoporosis, and have antimicrobial activities (11-13).

The Allium genus from the Amaryllidaceae family is rich in steroidal saponins, mainly concentrated in bulbs but also present in other tissues. Cultivated species typically contain spirostane and furostane types, while cholestane saponins occur in certain wild species (14, 15). Allium plants have been a staple of human nutrition since the time of ancient Egypt, the Persian Empire, and other civilizations (16, 17). Additionally, Allium species, as edible plants, provide valuable micronutrients that function as natural health supplements (18, 19).

*Allium jesdianum* Boiss. & Buhse, is an annual herbaceous species with bulbs, stems, and aerial parts growing 25 - 50 cm tall, bearing two to three leaves and spherical flowers ranging from pinkish to yellow-white (20-22).

Endemic to the Zagros Mountains of western Iran and eastern Iraq. The plant is locally known as “Bon-Sorkh” in Persian, “Bo-Sor” in Luri, and “Sourah-Boneh” in Kurdish. During early spring flowering, it is harvested by locals for market sale. In Lur and Kurd folk medicine, it has long been employed to treat kidney stones, rheumatoid arthritis, gastrointestinal disorders, and influenza (23-27).

**Figure 1.**
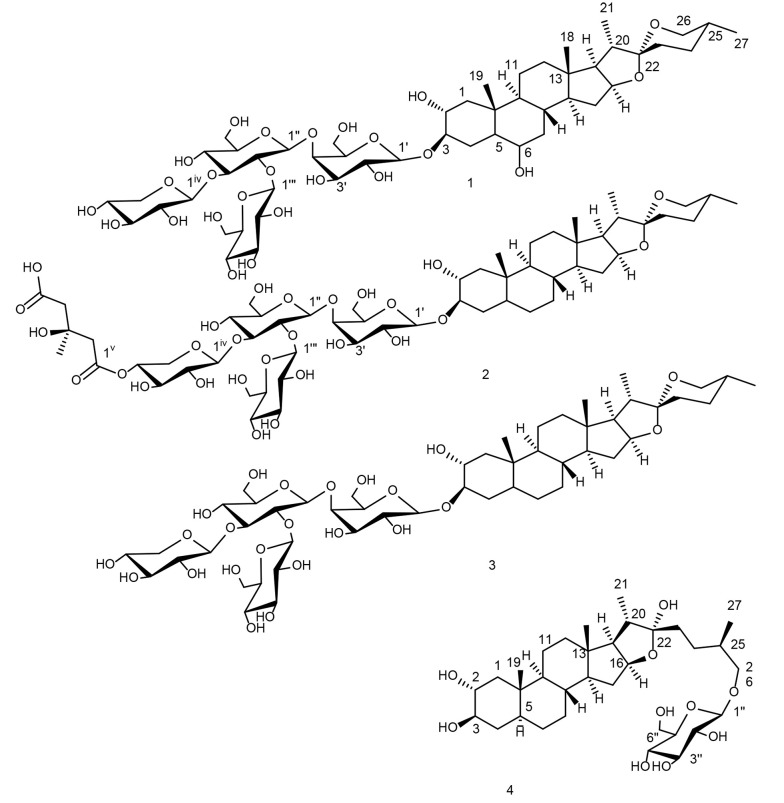
Saponins from *Allium jesdianum* aerial parts. Compound 1-3 were identified as (25R)-5α-spirostanol, and compound 4 as (25R)-5α-furostanol derivatives.

This study was carried out as part of the PhD research of one author (B.Z.), focusing on the extraction and characterization of saponins, and led to the discovery of potential saponins (Figure 1), compound 1, known as aginoside, found in *A. albopilosum* (28) and *A. schubertii*(29), 2 found in *Allium schubertii* and *A. cyrillii* (29, 30), 3, known as F-gitonin (23), and 4 found in *A. hirtifolium* and *A. chinensis* (31, 32).

We evaluated the antibacterial and antibiofilm effects of these compounds against colistin-resistant *K. pneumoniae*, *E. coli*, *A. baumannii*, and *P. aeruginosa*, using MIC and crystal violet assays. Gene expression of efflux pumps (RND family specifics) was further examined by real-time PCR following sub-MIC exposure. In addition, molecular docking was conducted to investigate the interaction of compound 3 with the AdeB efflux pump.

## 2. Methods

### 2.1. General Experimental

Electrospray ionization mass spectrometry (ESI-MS) was performed on an AB SCIEX QTRAP 3200 instrument (AB SCIEX, MA, USA) with methanol grade HPLC as solvent (Romil, Cambridge, UK). Nuclear magnetic resonance (NMR) spectra, including 1H (400.15 MHz) and 13C (100 MHz), were recorded on Bruker-400 spectrometers (Bruker, Karlsruhe, Germany). Thin-layer chromatography (TLC) was performed on SiO₂ plates (Merck, Frankfurt, Germany) with butanol–acetic acid–water (60:25:15) as the mobile phase, and cerium sulfate in 2N sulfuric acid (Qualigens Pharma, Mumbai, India) as the detection reagent. Also, anisaldehyde-sulfuric acid or vanillin-sulfuric acid can be an alternative reagent. Preparative HPLC was conducted using a Waters 600 pump (Waters Corp., MA, USA) in isocratic mode with a Waters refractive index detector and a YMC-Pack ODS column (5 µm, 250 × 20 mm; Kyoto, Japan) at a flow rate of 2 mL/min.

### 2.2. Plant Material

20 kg of aerial parts of *A. jesdianum* (fresh plants) were harvested in April 2022 during the flowering season from Sefidkuh Mountain, Khorramabad, Lorestan, Iran. Dr. Khatamsaz authenticated the specimen at Isfahan University of Medical Sciences, and a voucher sample (SAM-4244) was deposited in the Pharmacognosy Department of the same institution.

### 2.3. Extraction and Isolation

Air-dried aerial parts of *A. jesdianum* (Weight: 2900 g), were crushed and macerated sequentially at room temperature using hexanes, chloroform, chloroform: methanol (9:1), and methanol (each solvent: 3 days × 3 repetitions × 5 L). Chloroform: MeOH (9:1) extract afforded 40 g (labeled AJap-1), submitted on MPLC (C18 column, 36 × 460 mm) with gradient elution of MeOH: H2O (AJap-1a → 40:60; AJap-1b → 50:50; AJap-1c → 60:40; AJap-1d → 70:30; AJap-1e → 80:20; AJap-1f → 90:10; and AJap-1g → 100:0) with each step 500 mL. Based on preliminary ¹H-NMR analysis of all fractions, AJap-1f and AJap-1g were identified as saponin-rich fractions. For further purification, AJap-1f was submitted on HPLC using a YMC-ODS column (250 × 20 mm, 5 µm) under isocratic elution using H₂O: MeOH (20:80). The chromatogram was monitored by a refractive index (RI) detector, at a flow of 2 mL/min, resulting in compound 1 (16.2 mg) at Rt = 95 min. AJap-1g was purified by repeated precipitation of collection tubes 1 and 2 in pure MeOH by two times, resulting in compound 2 (32.31 mg) as a white solid. Similarly, AJap-2g from collection tubes 3 and 4 was purified and afforded compound 3 (76.2 mg). Methanol extract (yield: 56.5 g) was labeled as AJap-2 and was submitted to liquid-liquid partitioning between butanol and water in a separating funnel. Organic layer (BuOH layer) was concentrated (AJap-2a; 30 g), and fractionated on MPLC (C18 column, 36 × 460 mm) with gradient elution of MeOH: H₂O (AJap-2a-1 → 40:60; AJap-2a-2 → 50:50; AJap-2a-3 → 60:40; AJap-2a-4 → 70:30; AJap-2a-5 → 80:20; AJap-2a-6 → 90:10; and AJap-2a-7 → 100:0) with each fraction collected in 500 mL. After preliminary 1H-NMR screening of MPLC fractions, AJap-2a-5 with a saponin profile, was selected, and further purified on HPLC with the same system using H₂O: MeOH (30:70, isocratic), and afforded compound 4 (9.5 mg) with a retention time of 84 min.

### 2.4. Spectral Data of Isolated Compounds

Compound 1. White solid, yield: 16.2 mg. 1H-NMR: δH 1.72 (m, H-1a), 0.79 (m, H-1b), 3.63 (m, H-2), 3.25 (m, H-3), 1.89 (m, H-4a), 1.11 (m, H-4b), 1.05 (m, H-5), 3.13 (m, H-6) 1.65 (m, H-7a), 1.06 (m, H-7b), 1.78 (m, H-8), 0.67 (m, H-9), 1.45 (m, H-11a), 1.29 (m, H-11b), 1.81 (m, H-12a), 1.08 (m, H-12b), 1.09 (m, H-14), 1.60 (m, H-15a), 1.48 (m, H-15b), 4.25 (m, H-16), 1.67 (m, H-17), 0.72 (s, H-18), 0.91 (s, H-19), 1.82 (m, H-20), 0.88 (d, H-21), 1.61 (m, H-23a), 1.58 (m, H-23b), 1.59 (m, H-24a), 1.25 (m, H-24b), 1.52 (m, H-25), 3.40 (m, H-26a), 3.15 (m, H-26b), 0.73 (d, H-27), 4.23 (d, H-Gal1Ⅰ), 3.39 (m, H-Gal2Ⅰ), 3.06 (m, H-Gal3Ⅰ), 3.81 (m, H-Gal4Ⅰ), 3.40 (m, H-Gal5Ⅰ), 3.70 (m, H-Gal6Ⅰa), 3.42 (m, H-Gal6Ⅰb), 4.42 (d, H-Glc1Ⅱ), 3.56 (m, H-Glc2Ⅱ), 3.60 (m, H-Glc3Ⅱ), 3.33 (m, H-Glc4Ⅱ), 3.21 (m, H-Glc5Ⅱ), 3.71 (m, H-Glc6Ⅱa), 3.57 (m, H-Glc6Ⅱb), 4.72 (d, H-Glc1Ⅲ), 3.12 (m, H-Glc2Ⅲ), 3.16 (m, H-Glc3Ⅲ), 3.38 (m, H-Glc4Ⅲ), 3.15 (m, H-Glc5Ⅲ), 3.72 (m, H-Glc6Ⅲa), 3.38 (m, H-Glc6Ⅲb), 4.50 (d, H-Xyl1IV), 2.97 (m, H-Xyl2IV), 3.20 (m, H-Xyl3IV), 3.46 (m, H-Xyl4IV), 3.77 (m, H-Xyl5IVa), 3.07 (m, H-Xyl5IVb). 13C-NMR: 46.79 (C-1), 69.17 (C-2), 83.45 (C-3), 31.93 (C-4), 47.09 (C-5), 69.23 (C-6), 39.80 (C-7), 29.47 (C-8), 53.96 (C-9), 36.60 (C-10), 21.01 (C-11), 40.43 (C-12), 40.57 (C-13), 55.73 (C-14), 31.31 (C-15), 80.63 (C-16), 62.31 (C-17), 16.66 (C-18), 17.02 (C-19), 41.58 (C-20), 15.10 (C-21), 108.92 (C-22), 31.32 (C-23), 28.92 (C-24), 30.26 (C-25), 66.31 (C-26), 17.55 (C-27), 101.79 (C-1Ⅰ), 73.93 (C-2Ⅰ), 73.96 (C-3Ⅰ), 79.38 (C-4Ⅰ), 74.60 (C-5Ⅰ), 59.95 (C-6Ⅰ), 103.62 (C-Glc1Ⅱ), 79.80 (C-Glc2Ⅱ), 85.64 (C-Glc3Ⅱ), 69.88 (C-Glc4Ⅱ), 77.31 (C-Glc5Ⅱ), 61.49 (C-Glc6Ⅱ), 102.89 (C-Glc1Ⅲ), 70.34 (C-Glc2Ⅲ), 76.63 (C-Glc3Ⅲ), 71.43 (C-Glc4Ⅲ), 76.34 (C-Glc5Ⅲ), 61.72 (C-Glc6Ⅲ), 103.76 (C-Xyl1IV), 74.66 (C-Xyl2IV), 77.01 (C-Xyl3IV), 69.65 (C-Xyl4IV), 62.56 (C-Xyl5IV)). ESI-MS (Pos. ion) m/z = 1067.2 [M+H]+.

Compound 2. White solid, yield: 32.31 mg. 1H-NMR: δH 1.82 (m, H-1a), 0.81 (m, H-1b), 3.29 (m, H-2), 3.31 (m, H-3), 1.61 (m, H-4a), 1.25 (m, H-4b), 1.07 (m, H-5), 1.57 (m, H-6a), 1.31 (m, H-6b), 1.61 (m, H-7a), 0.87 (m, H-7b), 1.40 (m, H-8), 0.66 (m, H-9), 1.44 (m, H-11a), 1.24 (m, H-11b), 1.69 (m, H-12a), 1.08 (m, H-12b), 1.07 (m, H-14), 1.60 (m, H-15a), 1.47 (m, H-15b), 4.28 (m, H-16), 1.65 (m, H-17), 0.70 (s, H-18), 0.77 (s, H-19), 1.81 (m, H-20), 0.89 (d, H-21), 1.89 (m, H-23a), 1.15 (m, H-23b), 1.30 (m, H-24a), 1.17 (m, H-24b), 1.46 (m, H-25), 3.42 (m, H-26a), 3.20 (m, H-26b), 0.72 (d, H-27), 4.21 (d, H-Gal1Ⅰ), 3.39 (m, H-Gal2Ⅰ), 3.06 (m, H-Gal3Ⅰ), 3.82 (m, H-Gal4Ⅰ), 3.40 (m, H-Gal5Ⅰ), 3.72 (m, H-Gal6Ⅰa), 3.41 (m, H-Gal6Ⅰb), 4.45 (d, H-Glc1Ⅱ), 3.58 (m, H-Glc2Ⅱ), 3.62 (m, H-Glc3Ⅱ), 3.15 (m, H-Glc4Ⅱ), 3.25 (m, H-Glc5Ⅱ), 3.71 (m, H-Glc6Ⅱa), 3.57 (m, H-Glc6Ⅱb), 4.72 (d, H-Glc1Ⅲ), 3.15 (m, H-Glc2Ⅲ), 3.20 (m, H-Glc3Ⅲ), 3.39 (m, H-Glc4Ⅲ), 3.16 (m, H-Glc5Ⅲ), 3.71 (m, H-Glc6Ⅲa), 3.40 (m, H-Glc6Ⅲb), 4.51 (d, H-Xyl1IV), 2.99 (m, H-Xyl2IV), 3.36 (m, H-Xyl3IV), 4.56 (m, H-Xyl4IV), 3.88 (m, H-Xyl5IVa), 3.20 (m, H-Xyl5IVb), 1.26 (s, H-HMG1), 2.50 (m, H-HMG2a), 2.47 (m, H-HMG2b), 2.62 (m, H-HMG3a), 2.49 (m, H-HMG3b). 13C-NMR: 45.42 (C-1), 69.90 (C-2), 83.11 (C-3), 33.53 (C-4), 44.38 (C-5), 28.97 (C-6), 32.13 (C-7), 30.29 (C-8), 54.08 (C-9), 36.60 (C-10), 21.22 (C-11), 39.88 (C-12), 40.57 (C-13), 56.03 (C-14), 31.39 (C-15), 80.67 (C-16), 62.40 (C-17), 16.65 (C-18), 13.55 (C-19), 41.59 (C-20), 15.08 (C-21), 108.84 (C-22), 31.88 (C-23), 27.95 (C-24), 34.41 (C-25), 66.36 (C-26), 17.55 (C-27), 101.85 (C-1Ⅰ), 73.95 (C-2Ⅰ), 74.01 (C-3Ⅰ), 79.26 (C-4Ⅰ), 74.60 (C-5Ⅰ), 59.97 (C-6Ⅰ), 103.62 (C-Glc1Ⅱ), 79.86 (C-Glc2Ⅱ), 85.60 (C-Glc3Ⅱ), 69.27 (C-Glc4Ⅱ), 76.40 (C-Glc5Ⅱ), 61.50 (C-Glc6Ⅱ), 102.94 (C-Glc1Ⅲ), 70.35 (C-Glc2Ⅲ), 76.67 (C-Glc3Ⅲ), 71.48 (C-Glc4Ⅲ), 77.29 (C-Glc5Ⅲ), 61.76 (C-Glc6Ⅲ), 103.78 (C-Xyl1IV), 74.69 (C-Xyl2IV), 73.56 (C-Xyl3IV), 71.91 (C-Xyl4IV), 62.56 (C-Xyl5IV), 27.71 (C-HMG1), 46.02 (C-HMG2), 46.14 (C-HMG3), 69.50 (C-HMG4), 171.60 (C-HMG5), 173.01 (C-HMG6). ESI-MS (Pos. ion) m/z = 1079.2 [M-OH]+.

Compound 3. White solid, yield: 76.2 mg. 1H-NMR: δH 1.82 (m, H-1a), 0.82 (m, H-1b), 3.03 (m, H-2), 3.36 (m, H-3), 1.64 (m, H-4a), 1.22 (m, H-4b), 1.09 (m, H-5), 1.57 (m, H-6a), 1.23 (m, H-6b) 1.60 (m, H-7a), 0.86 (m, H-7b), 1.54 (m, H-8), 0.68 (m, H-9), 1.47 (m, H-11a), 1.25 (m, H-11b), 1.65 (m, H-12a), 1.09 (m, H-12b), 1.06 (m, H-14), 1.59 (m, H-15a), 1.48 (m, H-15b), 4.24 (m, H-16), 1.64 (m, H-17), 0.71 (s, H-18), 0.78 (s, H-19), 1.79 (m, H-20), 0.89 (d, H-21), 1.88 (m, H-23a), 1.11 (m, H-23b), 1.27 (m, H-24a), 1.18 (m, H-24b), 1.46 (m, H-25), 3.39 (m, H-26a), 3.18 (m, H-26b), 0.73 (d, H-27), 4.20 (d, H-Gal1Ⅰ), 3.34 (m, H-Gal2Ⅰ), 3.04 (m, H-Gal3Ⅰ), 3.79 (m, H-Gal4Ⅰ), 3.38 (m, H-Gal5Ⅰ), 3.70 (m, H-Gal6Ⅰa), 3.38 (m, H-Gal6Ⅰb), 4.41 (d, H-Glc1Ⅱ), 3.54 (m, H-Glc2Ⅱ), 3.59 (m, H-Glc3Ⅱ), 3.22 (m, H-Glc4Ⅱ), 3.22 (m, H-Glc5Ⅱ), 3.70 (m, H-Glc6Ⅱa), 3.56 (m, H-Glc6Ⅱb), 4.70 (d, H-Glc1Ⅲ), 3.12 (m, H-Glc2Ⅲ), 3.15 (m, H-Glc3Ⅲ), 3.34 (m, H-Glc4Ⅲ), 3.12 (m, H-Glc5Ⅲ), 3.54 (m, H-Glc6Ⅲa), 3.37 (m, H-Glc6Ⅲb), 4.48 (d, H-Xyl1IV), 2.96 (m, H-Xyl2IV), 3.13 (m, H-Xyl3IV), 3.44 (m, H-Xyl4IV), 3.75 (m, H-Xyl5IVa), 3.09 (m, H-Xyl5IVb). 13C-NMR: 45.40 (C-1), 69.89 (C-2), 83.04 (C-3), 33.51 (C-4), 44.40 (C-5), 28.93 (C-6), 32.14 (C-7), 30.26 (C-8), 54.05 (C-9), 36.76 (C-10), 21.19 (C-11), 39.85 (C-12), 40.56 (C-13), 55.98 (C-14), 31.37 (C-15), 80.64 (C-16), 62.37 (C-17), 16.64 (C-18), 13.54 (C-19), 41.56 (C-20), 15.07 (C-21), 108.86 (C-22), 31.85 (C-23), 27.94 (C-24), 34.40 (C-25), 66.36 (C-26), 17.54 (C-27), 101.79 (C-1Ⅰ), 73.93 (C-2Ⅰ), 73.97 (C-3Ⅰ), 79.23 (C-4Ⅰ), 74.58 (C-5Ⅰ), 59.95 (C-6Ⅰ), 103.58 (C-Glc1Ⅱ), 79.80 (C-Glc2Ⅱ), 85.59 (C-Glc3Ⅱ), 69.20 (C-Glc4Ⅱ), 76.35 (C-Glc5Ⅱ), 61.49 (C-Glc6Ⅱ), 102.89 (C-Glc1Ⅲ), 70.34 (C-Glc2Ⅲ), 76.63 (C-Glc3Ⅲ), 71.47 (C-Glc4Ⅲ), 77.26 (C-Glc5Ⅲ), 61.74 (C-Glc6Ⅲ), 103.76 (C-Xyl1IV), 74.66 (C-Xyl2IV), 77.02 (C-Xyl3IV), 69.59 (C-Xyl4IV), 66.37 (C-Xyl5IV). ESI-MS (Pos. ion) m/z = 1195.2 [M+H]+.

Compound 4. White solid, yield: 9.5 mg. 1H-NMR: δH 1.76 (m, H-1a), 0.80 (m, H-1b), 3.11 (m, H-2), 3.35 (m, H-3), 1.47 (m, H-4a), 1.25 (m, H-4b), 1.48 (m, H-5), 1.49 (m, H-6a), 1.24 (m, H-6b), 1.59 (m, H-7a), 1.07 (m, H-7b), 1.45 (m, H-8), 0.66 (m, H-9), 1.45 (m, H-11a), 1.22 (m, H-11b), 1.66 (m, H-12a), 1.08 (m, H-12b), 1.05 (m, H-14), 1.84 (m, H-15a), 0.85 (m, H-15b), 4.38 (m, H-16), 1.66 (m, H-17), 0.71 (s, H-18), 0.76 (s, H-19), 1.93 (m, H-20), 0.86 (d, J = 6.8 Hz, H-21), 1.57 (m, H-23a), 1.44 (m, H-23b), 1.48 (m, H-24a), 1.09 (m, H-24b), 1.61 (m, H-25), 3.53 (m, H-26a), 3.27 (m, H-26b), 0.85 (d, J = 6.4 Hz, H-27), 4.10 (d, J = 7.5 Hz, H-Glc1Ⅰ), 3.07 (m, H-Glc2Ⅰ), 3.09 (m, H-Glc3Ⅰ), 2.97 (m, H-Glc4Ⅰ), 3.13 (m, H-Glc5Ⅰ), 3.68 (m, H-Glc6Ⅰa), 3.43 (m, H-Glc-6Ⅰb). 13C-NMR: δC 45.93 (C-1), 75.63 (C-2), 71.98 (C-3), 36.64 (C-4), 44.72 (C-5), 28.05 (C-6), 32.04 (C-7), 34.43 (C-8), 54.30 (C-9), 37.14 (C-10), 21.23 (C-11), 40.09 (C-12), 40.90 (C-13), 56.01 (C-14), 32.23 (C-15), 80.24 (C-16), 63.01 (C-17), 16.81 (C-18), 13.67 (C-19), 40.08 (C-20), 16.24 (C-21), 110.07 (C-22), 36.20 (C-23), 27.75 (C-24), 33.54 (C-25), 74.26 (C-26), 17.54 (C-27), 103.37 (C-Glc1Ⅰ), 70.55 (C-Glc2Ⅰ), 77.22 (C-Glc3Ⅰ), 73.94 (C-Glc4Ⅰ), 77.25 (C-Glc5Ⅰ), 61.56 (C-Glc6Ⅰ). ESI-MS (Pos. ion) m/z = 613.1 [M+H]+.

### 2.5. Antimicrobial

#### 2.5.1. Microbial Isolation and Identification

For this study, Extended drug resistance isolates of *K. pneumoniae*, *E. coli*, *A. baumannii*, and *P. aeruginosa* were selected from patients admitted to hospitals, were obtained from patients admitted to hospitals, including the reference Al-Zahra Hospital (Isfahan, Iran), and identified according to previously established methods (e.g., API 20E) Isolates exhibiting both strong biofilm-forming capacity and XDR profiles were selected for further investigation.

#### 2.5.2. Antimicrobial Activity of Bioactive Compounds

The antimicrobial activity of the compounds was initially evaluated against *K. pneumoniae*, *E. coli*, *A. baumannii*, and *P. aeruginosa* using the agar well diffusion method. Freshly prepared bacterial cultures were adjusted to an OD600 of 0.1, suspended in sterile PBS, and 100 μL of each suspension was uniformly swabbed onto Mueller–Hinton agar plates. Wells of 6 mm diameter were cut into the agar, and 50 μL of each compound (1 - 4), dissolved in PBS at concentrations ranging from 4000 to 62.5 μg/mL, was added. Plates were incubated at 37 °C for 24 hours, after which inhibition zones surrounding the wells were measured to assess antimicrobial activity. A meropenem disc served as the positive control.

#### 2.5.3. Minimum Inhibitory Concentration of Bioactive Compounds

Minimum inhibitory concentrations (MICs) were determined using the broth microdilution method following the recommendations of the Clinical and Laboratory Standards Institute (CLSI) guidelines for antimicrobial susceptibility testing. For the MIC assay, overnight cultures of *K. pneumoniae*, *E. coli*, *A. baumannii*, and *P. aeruginosa* (10^8^ CFU/mL) were inoculated into 10 mL of Mueller–Hinton broth (MHB), and the OD600 was adjusted to 0.1. Subsequently, 100 μL of MHB was dispensed into each well of a 96-well polystyrene microtiter plate, followed by the addition of 100 μL of each compound (1-4), diluted to final concentrations ranging from 4000 to 62.5 μg/mL. For each compound, 5 μL of the bacterial suspension was then inoculated into the wells, and the plates were incubated at 37 °C for 24 hours. Broth medium alone served as the negative control, and all assays were performed in triplicate for each concentration.

#### 2.5.4. Biofilm Inhibitory Assay

To evaluate the antibiofilm activity of the extracts, 200 μL of Trypticase Soy Broth (TSB) was dispensed into each well of a 96-well polystyrene microtiter plate. Subsequently, 100 μL of each compound (1-4) was added and serially diluted to final concentrations ranging from 4000 to 62.5 μg/mL. Overnight cultures of the selected bacterial isolates were adjusted to 0.5 McFarland standard (≈10^8^ CFU/mL), and 5 μL of each suspension was inoculated into the wells. The bacterial inoculum was standardized to 0.5 McFarland (≈10^8^ CFU/mL) in accordance with CLSI antimicrobial susceptibility testing recommendations before inoculation into microtiter plates. Plates were incubated at 37 °C for 48 hours to allow biofilm formation. After incubation, the medium was discarded, and wells were gently washed with PBS (pH 7.2). Biofilms were fixed with 96% ethanol, stained with 0.1% crystal violet for 15 minutes, washed five times with distilled water, and subsequently solubilized in a 1:1 solution of 33% acetone and 80% ethanol. Biofilm biomass was quantified by measuring optical density at 600 nm (OD600) using an ELISA microtiter plate reader (Infinite F50, Tecan). All treatments were performed in triplicate. Broth medium with bacterial isolates served as the positive control, while broth without bacteria served as the negative control. The percentage of biofilm reduction was calculated using the following formula:


$$x=\frac{control\;untreated\;OD600\;nm\;-the\;mean\;of\;three\;replicate\;test\;OD600\;nm}{control\;untreated\;OD600\;nm}\;\times \;100$$

All OD600 values were normalized by subtracting the readings of stained, treated, and untreated (bacteria-only) samples from the OD600 of stained control wells containing bacteria-free medium.

#### 2.5.5. Quantitative Real-time PCR Analysis

Quantitative real-time PCR (qRT-PCR) was performed to evaluate the effect of compounds 1 - 4 on the expression of efflux pump and resistance genes (AdeB, AcrA, OprL, and BlaKPC) in *K. pneumoniae*, *E. coli*, *A. baumannii*, and *P. aeruginosa*. Overnight cultures of each isolate grown in Trypticase Soy Broth (TSB) were transferred to fresh TSB, treated with sub-MIC concentrations of the compounds, and incubated at 37 °C for 16 hours. Cells were then washed three times with sterile PBS (pH 7.2) and harvested by centrifugation at 4 °C for 10 minutes. Total RNA was extracted using an RNA extraction kit (Parstoos, Iran) according to the manufacturer’s instructions, followed by DNase I treatment to remove genomic DNA. Reverse transcription was carried out to synthesize cDNA using the Parstoos kit protocol. qRT-PCR was conducted on an ABI StepOne Plus™ system (Applied Biosystems, USA). Each 20 μL reaction contained 2× SYBR® Green Master Mix (Ampliqon, Denmark), diluted cDNA (5 ng/μL), primers (10 pM each), and RNase-free ddH2O. Thermocycling conditions included an initial denaturation at 95 °C for 10 minutes, followed by 40 cycles of denaturation at 95 °C for 15 seconds and annealing/extension at 60 °C for 60 seconds. 16S rRNA was used as the internal control. Primer sequences are listed in Supplemaentary File. All reactions were performed in triplicate, and relative gene expression was calculated using the 2^−ΔΔCT^ method. At the end of each amplification run, melt-curve analysis was performed to verify the specificity of PCR products and confirm the absence of non-specific amplification. Amplification efficiencies for each primer pair were determined from standard curves and are provided together with primer sequences in the Supplementary Information.

### 2.6. Docking

#### 2.6.1. Ligand Preparation

Molecular docking simulations were conducted using the Schrödinger Suite (Schrödinger Release 2015; Schrödinger LLC, New York, NY, USA). Accurate conversion of ligand structures from 2D to 3D, followed by structural optimization, is a critical prerequisite for molecular docking studies. Accordingly, the ligand was prepared using the MacroModel and LigPrep modules of the Schrödinger Suite 2015 (33, 34). Energy minimization was performed in the MacroModel environment employing the OPLS-AA 2005 force field, with solvent effects simulated using the generalized Born/surface area (GB/SA) solvation model and no cut-off for non-bonded interactions. The Polak–Ribiere conjugate gradient (PRCG) algorithm was applied, allowing up to 5000 iterations with a gradient convergence threshold of 0.001. Finally, the ligand was processed in LigPrep to ensure chemical accuracy and to generate the most probable ionization state at physiological pH = 7.4 ± 0.5 (35-37). This value was selected to provide a physiologically relevant protonation state for docking simulations.

#### 2.6.2. Protein Preparation

The crystal structure of the *Acinetobacter baumannii* AdeB efflux transporter (PDB ID: 6OWS) was retrieved from the RCSB Protein Data Bank (38). An appropriate initial structure for subsequent docking experiments was generated using the Protein Preparation Wizard (PPW) protocol in the Schrödinger Suite 2015 (34). Initially, the protein structure was preprocessed by removing non-essential components (including water molecules and maltose), adding hydrogen atoms, assigning bond orders, defining disulfide bonds, and reconstructing missing loops and side chains. Hydrogen bond optimization was then performed by predicting the protonation states of His, Asp, and Glu residues, rotating terminal groups of Asn, Gln, and His by 180°, and sampling thiol and hydroxyl hydrogens. Finally, energy minimization of the protein structure was carried out using the OPLS-2005 force field with an RMSD convergence threshold of 0.30 Å, yielding a refined receptor structure suitable for docking studies.

#### 2.6.3. Molecular Docking

Molecular docking simulations were conducted using the Schrödinger Suite 2015, applying the induced-fit docking (IFD) protocol as previously described (39, 40). This approach incorporates receptor flexibility by permitting structural adjustments within the active site, thereby improving ligand accommodation. Refinements such as side-chain repositioning and backbone relaxation enhance the accuracy of predicted ligand–binding configurations (41, 42).

The induced-fit docking (IFD) workflow comprised three sequential phases. First, ligands were positioned within a static receptor model using Glide’s standard precision (SP) mode, with van der Waals radii of non-polar atoms scaled to 0.5. Up to 20 potential binding orientations were retained for optimization. Based on the known location of the ligand within the AdeB binding domain, the grid box was centered at the inhibitor’s centroid (X = 34.124, Y = 16.298, Z = −58.712) to ensure precise coverage of the binding pocket, with dimensions configured to accommodate ligands up to ~20 A° in length. In the second phase, the Prime module refined the initial docking poses by allowing conformational flexibility in residues within 5 A° of each ligand, while keeping the remainder of the protein fixed. From this refinement, the ten receptor conformations with energies ≤30 kcal/mol above the lowest-energy structure were selected for high-precision re-docking using Glide XP mode under default parameters. Finally, complexes were ranked using the IFD score, which integrates ligand–receptor interaction energy and total system energy calculated with the OPLS-2005 force field. Visualizations of the docked complexes were generated using PyMOL and the Ligand Interaction diagrams available in Maestro (43).

#### 2.6.4. Statistical Analysis

Data are expressed as mean values with standard deviations from three independent experiments. Statistical differences among groups were analyzed using one-way ANOVA followed by Dunnett’s post hoc test in GraphPad Prism (version 8.0.1; GraphPad Software, CA, USA). Results with P < 0.05 were considered statistically significant.

## 3. Results

### 3.1. Phytochemical Analysis

Aerial parts of *Allium jesdianum* were sequentially extracted using hexane, chloroform, chloroform: MeOH (9:1), and MeOH. The chloroform: MeOH (9:1) extract was fractionated via MPLC and further purified by HPLC to yield compounds 1-3. The MeOH extract was partitioned between butanol and water, and the butanol-soluble fraction was subjected to chromatographic techniques, resulting in the isolation of one furostanol saponin (4). All four compounds were isolated for the first time from the aerial parts of this plant.

Compound 1 was isolated as a white amorphous solid (Yield: 16.2 mg, tR = 95 min). 13C-NMR showed 50 signals, 27 carbons as the main steroidal core, including four methyls, nine methylenes (one oxygenated at δC 66.31), eleven methins (four oxygenated at δC 69.17,69.23, 80.63, and 83.45), and three quaternary carbons, including one hemiketal carbon at δC 108.92, indicating spirostane-type aglycones (28). Sugar moiety contained 23 carbons, indicating three hexapyranose sugars and one pentapyranose, including four anomeric carbons at δC 101.79 (δH 4.23, m, Gal-1Ⅰ), 103.62 (δH 4.42, m, Glc-1Ⅱ) ppm, 102.89 (δH 4.72, m, Glc-1Ⅲ), and 103.76 (δH 4.50, m, Xyl-1Ⅳ). ¹H NMR showed characteristic signals for four methyl groups, including two singlets at δH 0.72 (H3-18) and 0.91 (H3-19), and two doublets at δH 0.88 (d, J=6.8 Hz, H3-21) and 0.73 (d, J=6.4 Hz, H3-27). HMBC allowed to assign the spirostanol aglycone part as 5α-spirostane-2α,3β, 6β-triol (28). HSQC-TOCSY confirmed the identity and configuration of all four sugar units. HMBC of Gal H-1I ↔ C-3, and NOESY of Gal H-1I ↔ H-3, HMBC of Glc H-1Ⅱ ↔ C-4I, HMBC of Glc H-1Ⅲ ↔ C-2II, and HMBC of Xyl H-1Ⅳ ↔ C-3II determined glycosylation sites and linkages (23, 28, 29). HMBC of H₃-18 with C-12, C-13, C-14, C-17, and C-10; H3-27 with C-23, C-24, and C-26; H3-19 with C-1, C-9, and C-10; H3-21 with C-17, C-20, and C-22; H-17 with C-20, and C-21; H-20 with C-15, C-17, and C-22, H-26 with C-22; Gal-H11 with C-3; allowed to connect substructures, terminal methyls, and quaternary carbons to confirm and assign the spirostanol saponin. HMBC allowed to assign the spirostanol aglycone part as 5α-spirostane-2α,3β, 6β-triol (28). 2D HSQC and HSQC-TOCSY identified the sequence of four sugar spin systems as one galactose, two glucose, and one xylose unit. HMBC of Gal-H-1I with C-3, and NOESY of H-1I with H-3 determined GalⅠ linkage to C-3 of aglycone. HMBC of Glc-H-1Ⅱ with Gal-C-4I. Other glycosylation linkages for GlcII, GlcⅢ, and XylⅣ were determined through HMBC of H-1Ⅱ with C-4I, H-1Ⅲ with C-2II, and H-1Ⅳ with C-3II. The structure was determined as (25R)-5α-spirostan-2α,3β,6β-triol 3-O-β-D-glucopyranosyl-(1→2)-O-[β-D-xylopyranosyl-(1→3)]-O-β-D-glucopyranosyl-(1→4)-β-D-galactopyranoside, known as aginoside, in agreement with the literature (Figure 1) (23, 28, 29).

Compound 2 (530 mg), exhibited 56 distinct 13C-NMR signals. These comprised 27 carbons of the steroidal backbone, including four methyl groups, ten methylenes (one oxygenated at δC 66.36), ten methines (three oxygenated at δC 69.89, 80.64, and 83.08), and three quaternary carbons. Notably, a hemiketal carbon resonating at δC 108.84 ppm confirmed the presence of spirostane-type aglycones (29). Sugar moiety contained 23 carbons, indicating three hexa-pyranose sugars, and one penta-pyranose with four anomeric carbons at δC: 101.82, 103.60, 102.91, and 103.78 ppm. One HMG unit was seen with one methyl linkage with quaternary carbon at δC: 27.71, two methylenes, two carboxylic acid groups at δC: 170.56 and 172.97ppm (30). ¹H-NMR showed characteristic signals of four methyl groups, including two singlets at δH 0.69 (H3-18), and 0.76 (H3-19), and two doublets at δH 0.88 (d, J= 6.9 Hz, H3-21), and 0.70 (d, J= 6.3 Hz, H3-27), and one methyl from HMG at δH: 1.27 (H6-HMGⅤ). Anomeric protons showed signals at δH: 4.19 (d, J = 7.7 Hz, Gal-1Ⅰ), 4.42 (d, J = 7.8 Hz, Glc-1Ⅱ), 4.70 (d, J = 7.8 Hz, Glc-1Ⅲ), and 4.42 (d, J = 7.8 Hz, Xyl-1Ⅳ). 2D-NMR analysis of the aglycone part assigns the spirostanol aglycone part similar to that reported by N. V. Tolkacheva for 5-alpha-spirostane-2alpha,3beta diol (30). 2D HSQC and HSQC-TOCSY identified the sequence of four sugar spin systems as one galactose, two glucose, and one xylose unit. HMBC of Gal-H-1I with C-3, and ROESY of H-1I with H-3 determined GalⅠ linkage to C-3 of aglycone. HMBC of Glc-H-1Ⅱ with Gal-C-4I. Other glycosylation linkages for GlcII, GlcⅢ, Xyl Ⅳ, and HMGⅤ were determined through HMBC of H-1Ⅱ with C-4I, H-1Ⅲ with C-2II, H-1Ⅳ with C-3II, and H-3Ⅳ with C-1Ⅴ. Finally structure was assigned as (25R)-5α-spirostan-2α,3β-diol 3-O-β-D-glucopyranosyl-(1→2)-O-[4-O-(3S)-3-hydroxy-3-methylglutaroyl-β-D-xylopyranosyl-(1→3)]-O-β-D-glucopyranosyl-(1→4)-β-D-galactopyranoside, named as F-gitonin B in agreement with the literature (Figure 1) (30).

Compound 3 (76.2 mg) was afforded with a molecular formula of C50H82O23. Its 13C-NMR showed 50 signals, including 27 carbons as the main steroidal core, including four methyls, ten methylenes (one oxygenated at δC 66.36), ten methins (three oxygenated at δC 69.89, 80.64, and 83.04), and three quaternary carbons, including one hemiketal carbon at δC 108.86, indicating spirostane-type aglycones. Sugar moiety contained 23 carbons, indicating three hexapyranose sugars and one pentapyranose, with four anomeric carbons at δC 101.79 (δH 4.20, m, Gal-1Ⅰ), 103.58 (δH 4.41, m, Glc-1Ⅱ) ppm, 102.89 (δH 4.70, J=7.7 Hz, Glc-1Ⅲ), and 103.76 (δH 4.48, m, Xyl-1Ⅳ). ¹H NMR showed characteristic signals of four methyl groups, including two singlets at δH 0.71 (H3-18) and 0.78 (H3-19), and two doublets at δH 0.89 (d, J=6.7 Hz, H3-21) and 0.73 (d, J=6.0 Hz, H3-27). HMBC correlated partial substructures and assigned the aglycone as 5α -spirostane-2α, 3β-diol. HSQC-TOCSY confirmed the identity and configuration of all four sugar units. HMBC of Gal H-1I ↔ C-3, and NOESY of H-1I ↔ H-3, HMBC of Glc H-1Ⅱ ↔ C-4I, HMBC of Glc H-1Ⅲ ↔ C-2II, and HMBC of Xyl H-1Ⅳ ↔ C-3II determined glycosylation sites and linkages(23, 44, 45). HMBC of H₃-18 with C-12, C-13, C-14, C-17, and C-10; H3-27 with C-23, C-24, and C-26; H3-19 with C-1, C-9, and C-10; H3-21 with C-17, C-20, and C-22; H-17 with C-20, and C-21; H-20 with C-15, C-17, and C-22, H-26 with C-22; Gal-H11 with C-3; allowed to connect substructures, terminal methyls, and quaternary carbons to confirm and assign the spirostanol saponin (23, 44, 45). 2D HSQC and HSQC-TOCSY identified the sequence of four sugar spin systems as one galactose, two glucose, and one xylose unit. HMBC of Gal-H-1I with C-3, and NOESY of H-1I with H-3 determined GalⅠ linkage to C-3 of aglycone. HMBC of Glc-H-1Ⅱ with Gal-C-4I. Other glycosylation linkages for GlcII, GlcⅢ, and XylⅣ were determined through HMBC of H-1Ⅱ with C-4I, H-1Ⅲ with C-2II, H-1Ⅳ with C-3II (23, 44, 45). Finally, compound 3, as (25R)-5α-spirostan-2α,3β-diol 3-O-β-D-glucopyranosyl-(1→2)-O-[β-D-xylopyranosyl-(1→3)]-O-β-D-glucopyranosyl-(1→4)-β-D-galactopyranoside, was obtained for the first time from the aerial part of this plant, named as F-gitonin A, and in agreement with previous data reported by Kawashima (Figure 1) (23, 44, 45).

Compound 4 was obtained as a white solid (38.8 mg) with the molecular formula C₃₃H₅₆O₁₀. The 13C-NMR spectrum displayed 33 signals, including 27 carbons of the steroidal core: four methyl groups, ten methylenes (one oxygenated at δC 74.33), ten methines (three oxygenated at δC 71.94, 75.60, and 80.21), and three quaternary carbons, among which a hemiketal carbon resonated at δC 110.03 ppm. The sugar moiety comprised six carbons, consistent with a glucopyranose unit, observed at δC 103.33 (C-1Ⅰ), 70.52 (C-2Ⅰ), 77.20 (C-3Ⅰ), 73.91 (C-4Ⅰ), 77.23 (C-5Ⅰ), and 61.51 (C-6Ⅰ). ¹H-NMR showed characteristic signals of four methyl groups, including two singlets at δH: 0.69 (H3-18), and 0.74 (H3-19), and two doublets at δH: 0.88 (d, J = 6.8 Hz, H3-21), and 0.82 (d, J = 6.4 Hz, H3-27) as well as one anomeric signal at δH: 4.09 (d, J = 7.5 Hz) ppm. HMBC (H-1′ of sugar unit↔ C-26 (δC: 74.33 ppm) of aglycone) located Glc at C-26. It was identified as (25R)-5α-furostane-2a,3β,22α,26-tetraol-26-O-β-D-glucopyranoside, named as hirtifolioside C1 for the first time from this plant, in agreement with the literature (31, 32).

### 3.2. Antimicrobial

#### 3.2.1. Antimicrobial activity and Minimum Inhibitory Concentration of compounds

The antibacterial properties of all compounds were evaluated using the agar well diffusion method against four clinically relevant bacterial strains: *K. pneumoniae*, *E. coli*, *A. baumannii*, and *P. aeruginosa*. Across all tested concentrations and species, none of the compounds exhibited bactericidal activity after 18 hours of incubation, as evidenced by the absence of inhibition zones. Despite the lack of bactericidal effect, all compounds demonstrated measurable inhibitory activity in MIC assays.

Compounds 1 - 3 consistently displayed lower MIC values (4 mg/mL) relative to compound 4, reflecting enhanced antibacterial activity. The MIC profiles against the tested strains are presented in Table 1. Although none of the compounds reached bactericidal levels under the experimental conditions, compounds 1 - 3 exhibited stronger growth inhibition, underscoring the need for further studies on their mechanisms of action and therapeutic potential.

The relatively high MIC concentrations, together with the absence of bactericidal zones in agar diffusion assays, suggest that the isolated saponins are unlikely to act as standalone antibacterial agents. Rather, their relevance appears to lie in combination therapy with standard antimicrobial agents, particularly through mechanisms such as biofilm, rather than through strong direct antimicrobial effects.

**Table 1.** Minimum Inhibitory Concentration of Bioactive Compounds

Colistin-Resistant Bacterial Strain	Compound 1	Compound 2	Compound 3	Compound 4	Colistin
***K. pneumoniae***	4	4	4	8	16
***E. coli***	4	4	4	8	16
***A. baumannii***	4	4	4	8	32
***P. aeruginosa***	4	4	4	8	32

#### 3.2.2. Biofilm Reduction

**Figure 2.**
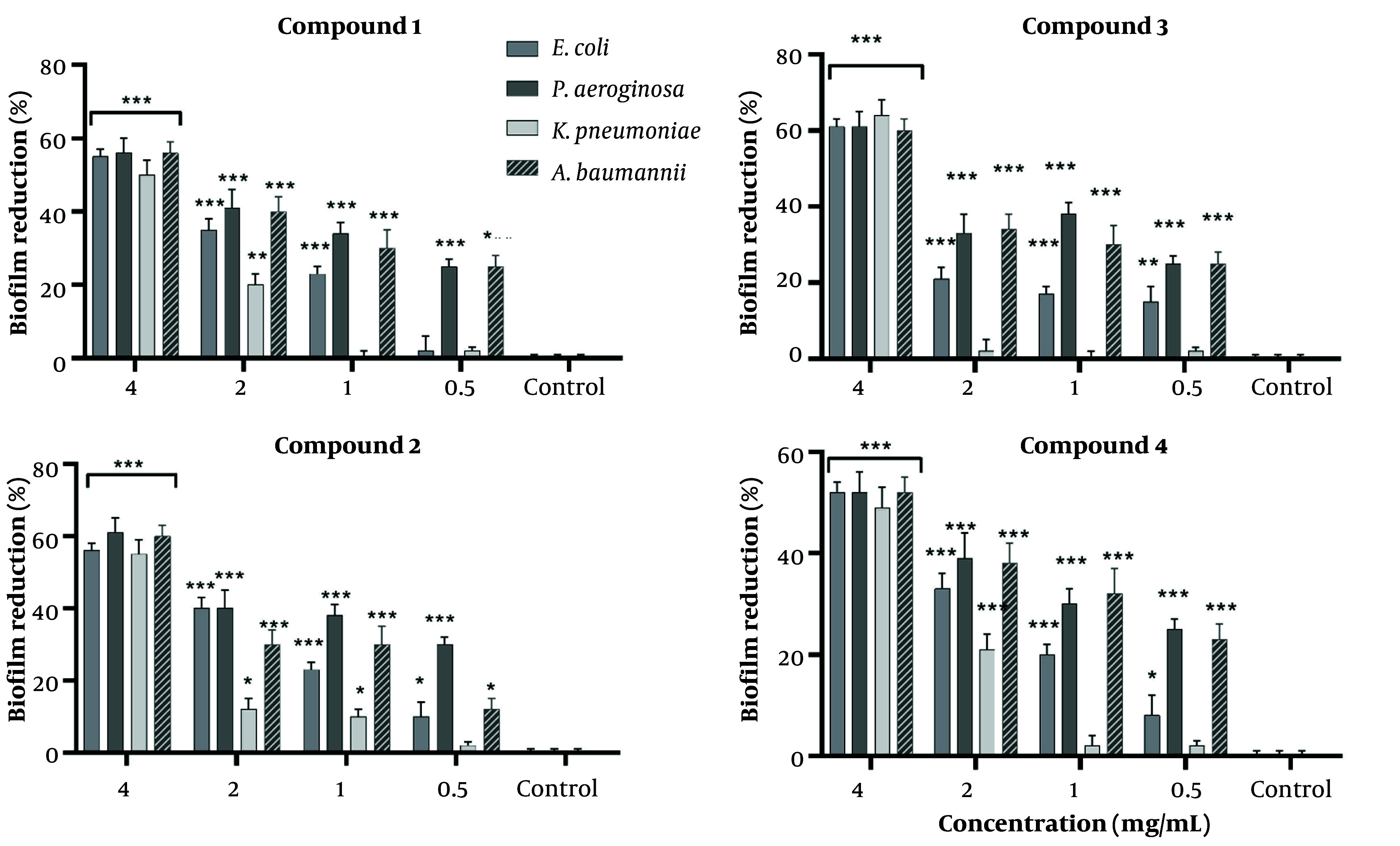
Biofilm reduction by Compounds 1 - 4 against selected bacterial pathogens.

The biofilm reduction assay showed concentration-dependent effect for all compounds against the tested bacterial strains (Figure 2). Biofilm reduction assays revealed that Compound 3 exhibited the highest inhibitory activity across all tested strains, with reductions approaching 70% - 75% against *E. coli*, *P. aeruginosa*, *K. pneumoniae* and A. Baumannii at 4 mg/mL. Compounds 1 and 2 exhibited moderate and comparable activity, typically reducing biofilm formation by approximately 40% - 60% at the highest concentration, depending on the strain. Compound 4 demonstrated the weakest performance. All compounds displayed a concentration-dependent decline in activity; however, Compounds 3 retained moderate efficacy against *P. aeruginosa* and *A. baumannii*, even at 0.5 mg/mL, suggesting potential for low-dose applications (P < 0.01). These findings highlight compound 3 for further development as an antibiofilm agent (Figure 2).

Compounds were tested at concentrations of 4, 2, 1, and 0.5 mg/mL against *E. coli*, *P. aeruginosa*, *K. pneumoniae*, and *A. baumannii*. Biofilm biomass was quantified using crystal violet staining after 48 h of incubation. Percent reduction was calculated relative to untreated bacterial controls. Data represent mean values from triplicate experiments.

#### 3.2.3. Quantitative Real-Time Polymerase Chain Reaction analysis

**Figure 3.**
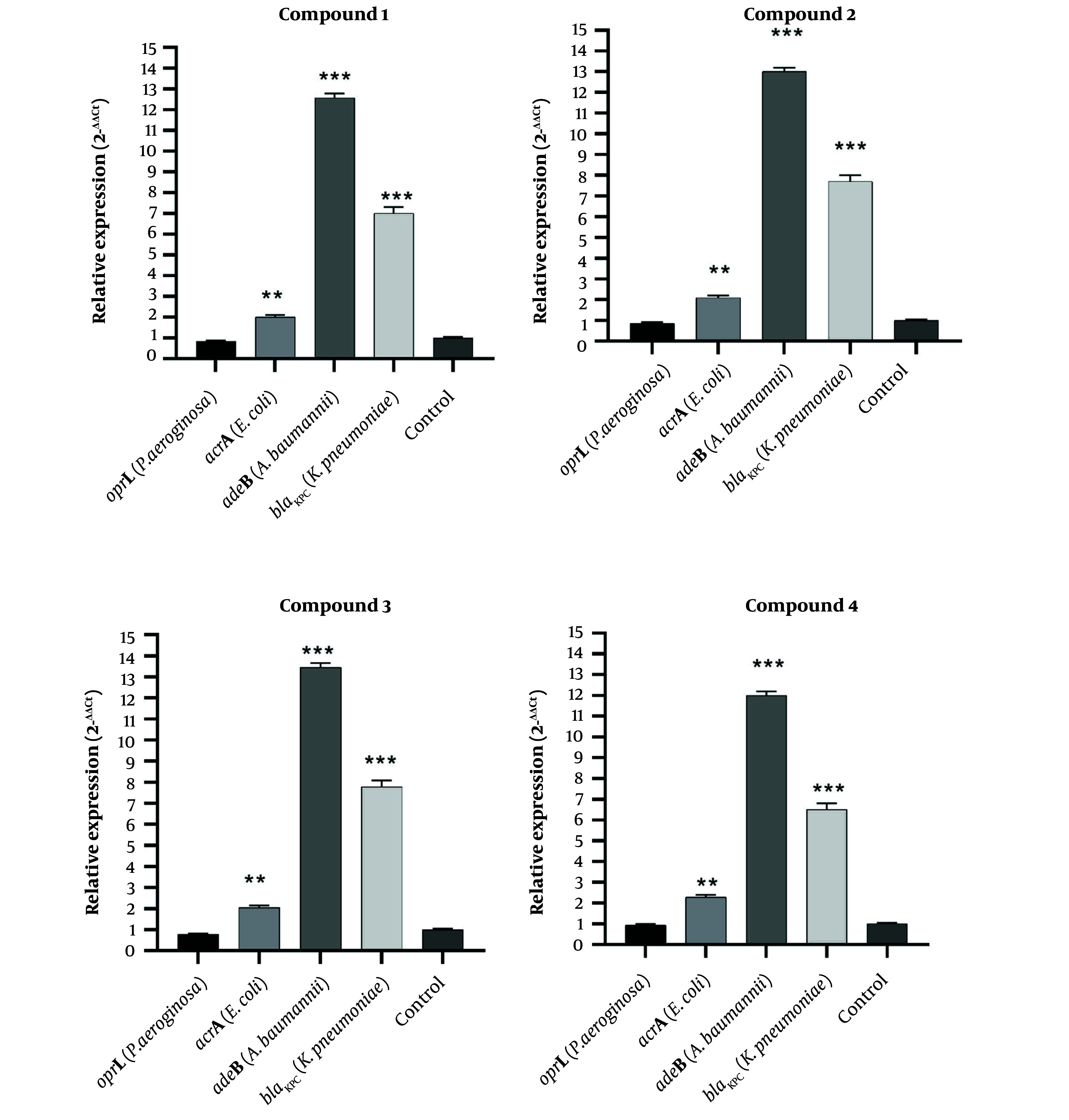
Gene expression modulation by compounds 1 - 4. Relative expression of resistance genes AdeB, AcrA, OprL, and BlaKPC in *K. pneumoniae*, *E. coli*, *A. baumannii*, and *P. aeruginosa* following sub-MIC treatment. Data were normalized to 16S rRNA and expressed as mean 2−ΔΔCT ± SD (n=3). Significance versus untreated control: *P < 0.05, **P < 0.01, ***P < 0.001.

Among the tested derivatives, compound 3 elicited the most pronounced induction of efflux-associated genes, with AdeB in *A. baumannii* showing the highest response (14.5-fold), accompanied by BlaKPC in *K. pneumoniae* (10.5-fold), AcrA in *E. coli* (7.5-fold), and OprL in *P. aeruginosa* (4.5-fold) (P < 0.05). In contrast, compound 2 primarily enhanced BlaKPC expression in *K. pneumoniae* (13.5-fold), while stimulating adeB in *A. baumannii* (10.5-fold), AcrA in *E. coli* (7.0-fold), and OprL in *P. aeruginosa* (4.0-fold). Compound 1 caused BlaKPC activation in *K. pneumoniae* (12.5-fold), with induction of AdeB in *A. baumannii* (9.5-fold), AcrA in *E. coli* (6.5-fold), and OprL in *P. aeruginosa* (3.5-fold). Finally, compound 4 expressed BlaKPC in *K. pneumoniae* upregulated by ~12.5-fold, AdeB in *A. baumannii* by ~9.0-fold, AcrA in *E. coli* by ~6.0-fold, and oprL in *P. aeruginosa* by ~3.0-fold (Figure 3).

Although compounds 1 - 3 exhibited measurable antimicrobial activity (MIC = 4 mg/mL) and notable antibiofilm effects, the overall antibacterial potency remained relatively weak. The qRT PCR analysis provides a clarification for this observation. All tested compounds induced the expression of efflux-associated resistance genes, indicating that bacterial cells responded to compound exposure by activating active drug export mechanisms. Efflux pump upregulation can reduce intracellular accumulation of antimicrobial agents, thereby limiting their bacteriostatic or bactericidal efficacy.

### 3.3. Molecular Docking

Because of greater upregulation of AdeB expression in *A. baumannii* upon treatment with compound 3, its molecular docking studies were done to elucidate its XP Glide scores, and Induced Fit Docking (IFD) results within the active site of the AdeB efflux pump (Table 2). Structural analysis revealed that the AdeB protein possesses two primary binding sites: a proximal pocket and a distal cavity, the latter also known as the deep binding pocket (DBP). The DBP plays a critical role in the antibiotic extrusion process mediated by the bacterial efflux pump. This cavity is notably characterized by a cluster of phenylalanine residues and other hydrophobic amino acids, which are collectively known as the hydrophobic trap. The hydrophobic nature of the AdeB binding cavity has been recognized as a major determinant influencing its interaction with various EPIs (38, 46).

**Figure 4.**
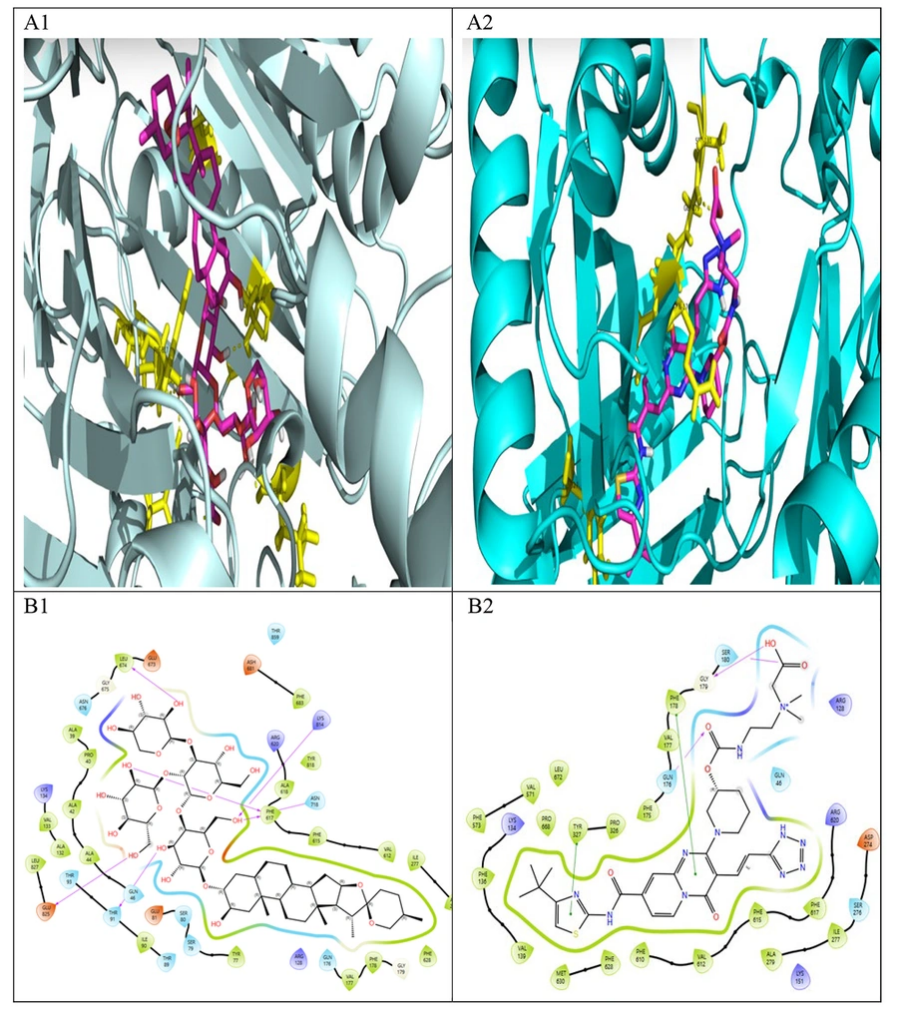
Induced-fit docking results for Compound 3 (A1, B1) and the reference inhibitor (A2, B2) in the AdeB Deep Binding Pocket (DBP). A1, A2: 3D binding poses showing ligands (ball-and-stick) and key interacting residues (sticks). B1, B2: 2D interaction diagrams detailing hydrogen bonds, hydrophobic contacts, and π-stacking.

Compound 3 exhibited marked binding affinity to the AdeB efflux pump, with an XP Glide score of –18.358 kcal/mol, compared to –13.357 kcal/mol for the reference. Both ligands were predicted to bind within the DBP. Hydrogen bonding analysis revealed that Compound 3 formed key interactions with Thr91, Asn718, and Glu825, and involved in hydrophobic and π–π interactions with Phe617 and Lys814 (Table 2, Figure 4). The IFD score of Compound 3 was –2045.77 kcal/mol comparable with the reference by -2053.72 kcal/mol. The docking score of -18.358 kcal/mol suggests strong predicted binding, even surpassing the reference ligand. It is in agreement with published docking scores of established efflux pump inhibitors (EPIs), such as PAβN or reserpine, which typically fall within a similar energetic range.

**Table 2.** Results of Induced Fit Docking Studies

Compound	XP Glide Score (kcal/mol)	IFD Score (kcal/mol)	H-Bond	Hydrophobic	π-π Interaction
**Compound 3**	-18.358	-2045.77	Thr91, Phe617, Asn718, Lys814, Glu825	Phe617, Lys814, Thr91	Phe617
**Reference**	-13.357	- 2053.72	Gln176, Gly179, Ser180	Phe178, Tyr327	Phe178, Tyr327

## 4. Conclusion

This study successfully isolated four known steroidal saponins from the aerial parts of *Allium jesdianum*, expanding the phytochemical profile of this species. While the isolated compounds exhibited weak direct antibacterial activity (MIC = 4 mg/mL), however, molecular investigations revealed a dual antibacterial mechanism. They demonstrated significant concentration-dependent antibiofilm effects, particularly compound 3, which reduced biofilm formation (up to 75% reduction at 4 mg/mL). The qRT PCR analysis provides a clarification for weak antimicrobial observation. All compounds induced the overexpression of key efflux pump genes (AdeB, AcrA, BlaKPC, and OprL), with compound 3 eliciting more transcriptional response in *A. baumannii*. It indicates that bacterial cells responded to compound exposure by activating active drug export mechanisms leading to reduction of intracellular antimicrobial agents, and limiting their bacteriostatic or bactericidal efficacy. In silico docking supported these findings, showing compound 3 to interact properly with the AdeB efflux pump binding pocket. Future in vivo studies on these saponins in combination with standard antibacterial agents are required for improvement of these studies.

ijpr-25-1-169955-s001.pdf
