# Supplementary material for: Four Steroidal Saponins Isolated from the Aerial Parts of Allium jesdianum Exhibit Antibiofilm Effects Against Colistin-Resistant Clinical Strains, with an In Silico Study
Source: Iran J Pharm Res. 2026 May 4;25(1):e169955. doi: 10.5812/ijpr-169955 (PMC13187685; doi:10.5812/ijpr-169955)
Supplement: ijpr-25-1-169955-s001.pdf [file ijpr-25-1-169955-s001.pdf]

Supplementary Information

Associated Article: Four saponins isolated from the aerial parts of *Allium jesdianum* exhibit antimicrobial effects against clinical strains resistant to Colistin, with in silico study

Contents:

Figure S1. 1H NMR (400 MHz, DMSO-d6) spectrum of compound 1.

Figure S2. 13C NMR (100 MHz, DMSO-d6) spectrum of compound 1.

Figure S3. DEPT-90 spectrum of compound 1.

Figure S4. DEPT-135 spectrum of compound 1.

Figure S5. HSQC spectrum of compound 1.

Figure S6. HMBC spectrum of compound 1.

Figure S7. HSQC-TOCSY spectrum of compound 1.

Figure S8. Positive ESI Mass spectrum of compound 1

Figure S9. 1H-NMR (400 MHz, DMSO-d6) spectrum of compound 2.

Figure S10. 13C-NMR (100 MHz, DMSO-d6) spectrum of compound 2.

Figure S11. DEPT-90 spectrum of compound 2.

Figure S12. DEPT-135 spectrum of compound 2.

Figure S13. HSQC spectrum of compound 2.

Figure S14. HMBC spectrum of compound 2.

Figure S15. HSQC-TOCSY spectrum of compound 2.

Figure S16. Positive ESI Mass spectrum of compound 2

Figure S17. 1H NMR (400 MHz, DMSO-d6) spectrum of compound 3.

Figure S18. 13C NMR (100 MHz, DMSO-d6) spectrum of compound 3.

Figure S19. DEPT-90 spectrum of compound 3.

Figure S20. DEPT-135 spectrum of compound 3.

Figure S21. HSQC spectrum of compound 3.

Figure S22. HMBC spectrum of compound 3.

Figure S23. HSQC-TOCSY spectrum of compound 3.

Figure S24. Positive ESI Mass spectrum of compound 3

Figure S25. 1H NMR (400 MHz, DMSO-d6) spectrum of compound 4.

Figure S26. 13C NMR (100 MHz, DMSO-d6) spectrum of compound 4.

Figure S27. DEPT-90 spectrum of compound 4.

Figure S28. DEPT-135 spectrum of compound 4.

Figure S29. HSQC spectrum of compound 4.

Figure S30. HMBC spectrum of compound 4.

Figure S31. HSQC-TOCSY spectrum of compound 4.

Figure S32. Positive ESI Mass spectrum of compound 4

Table S1. The list of primers sequences for real-time PCR

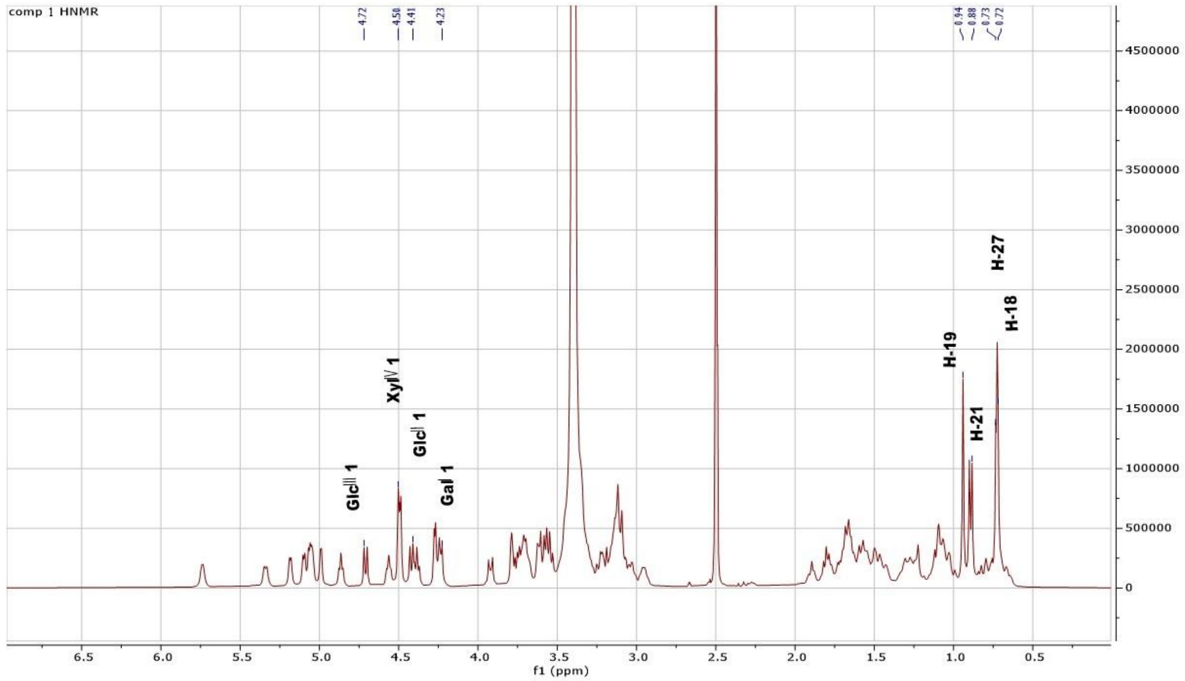

Figure S1. 1H NMR (400 MHz, DMSO-d6) spectrum of compound 1.

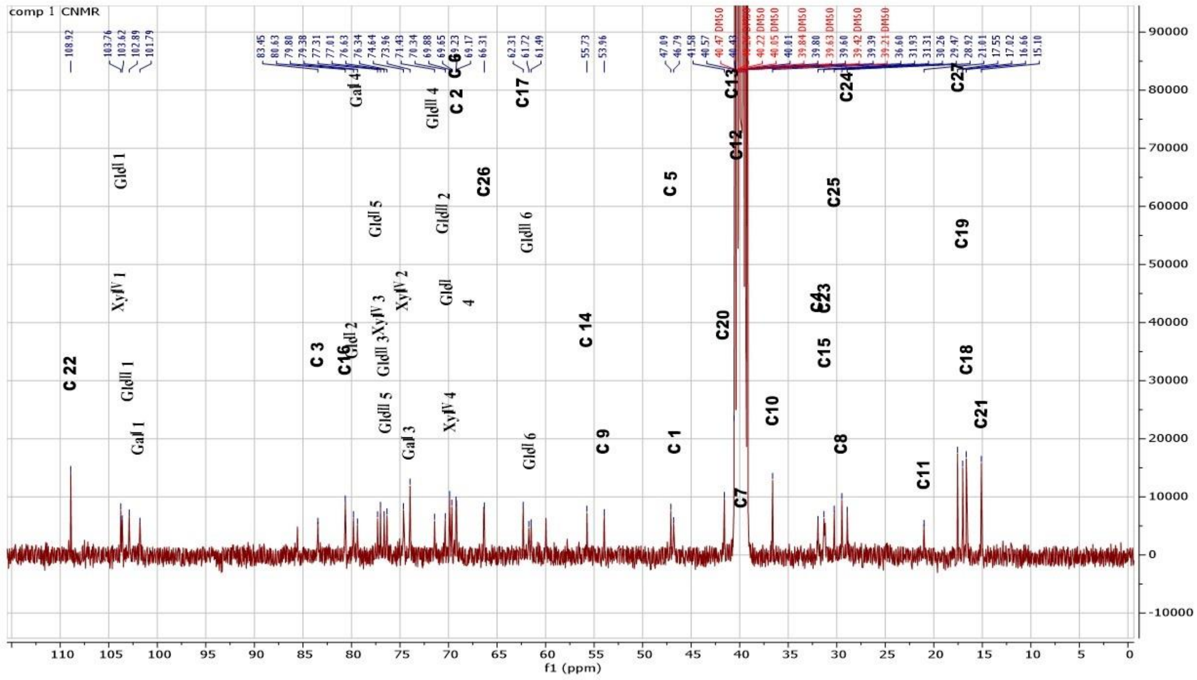

Figure S2. 13C NMR (100 MHz, DMSO-d6) spectrum of compound 1.

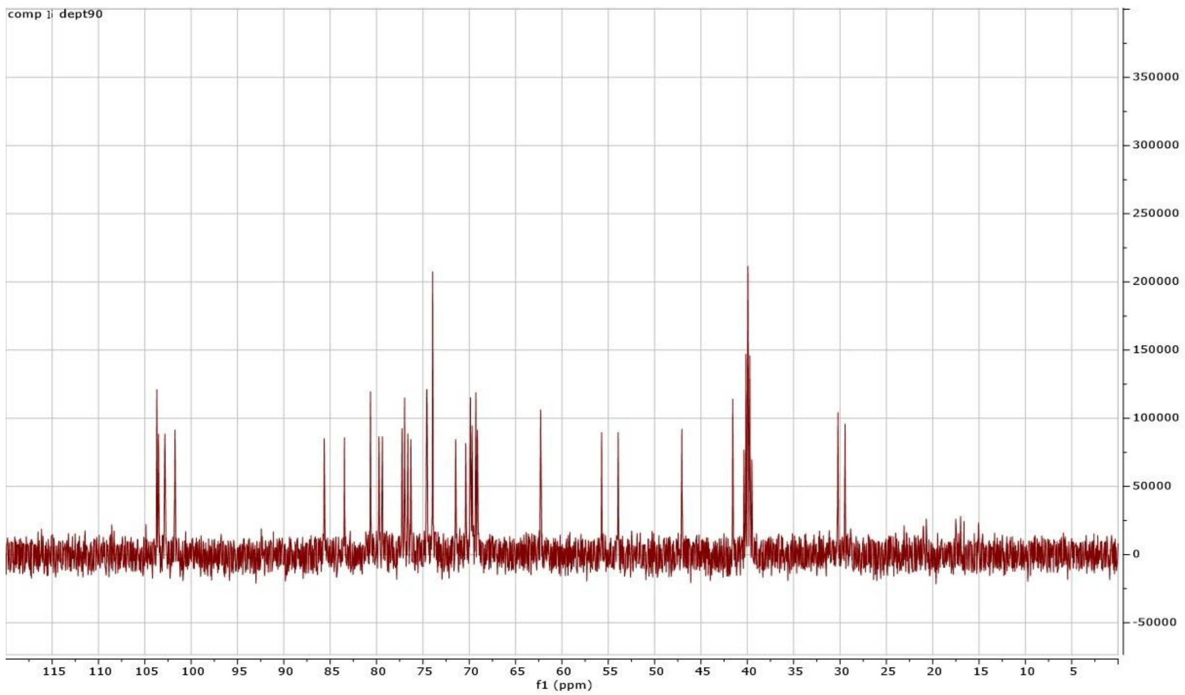

Figure S3. DEPT-90 spectrum of compound 1.

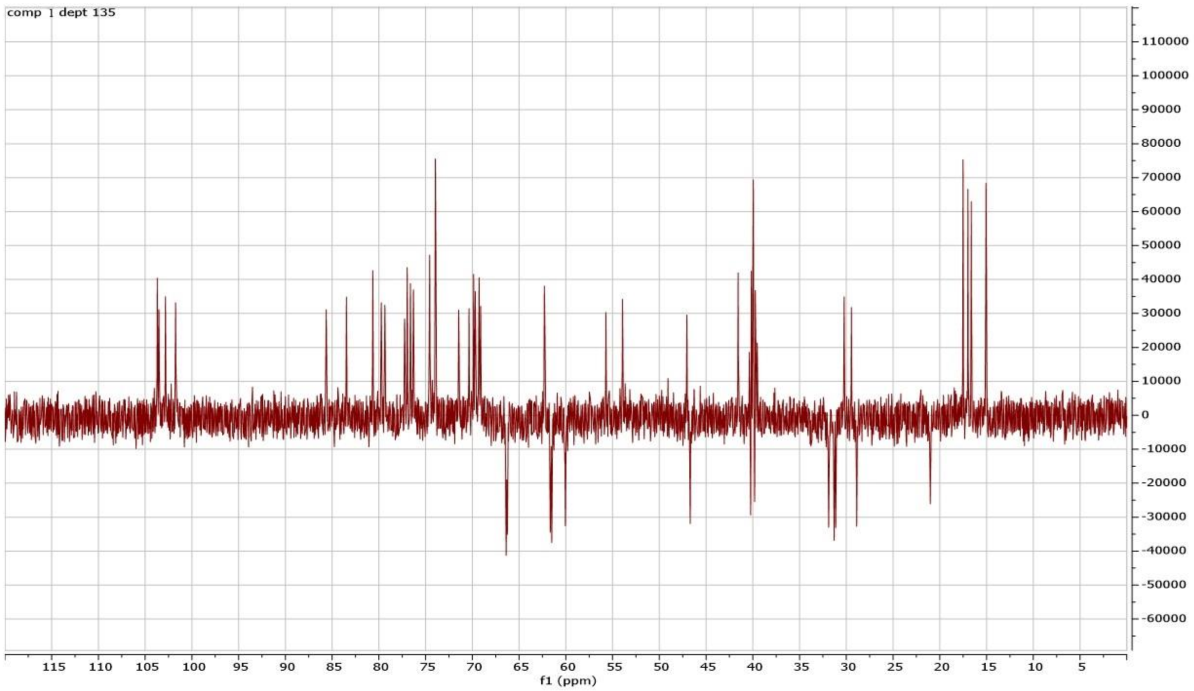

Figure S4. DEPT-135 spectrum of compound 1.

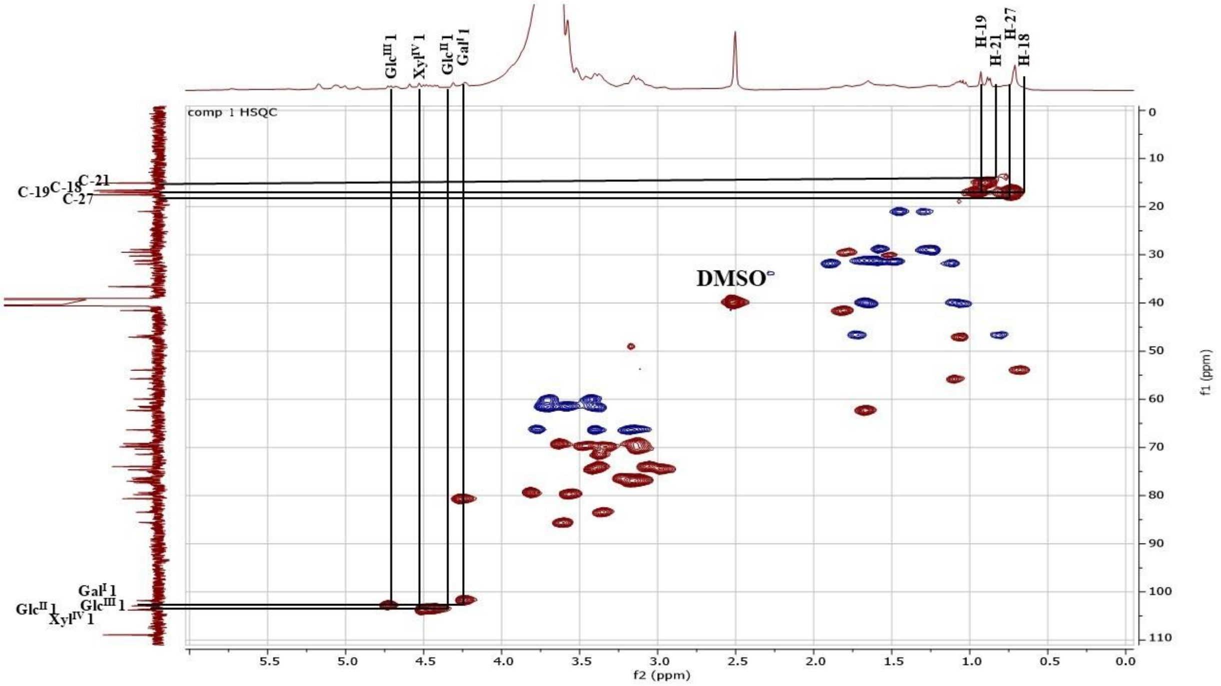

Figure S5. HSQC spectrum of compound 1.

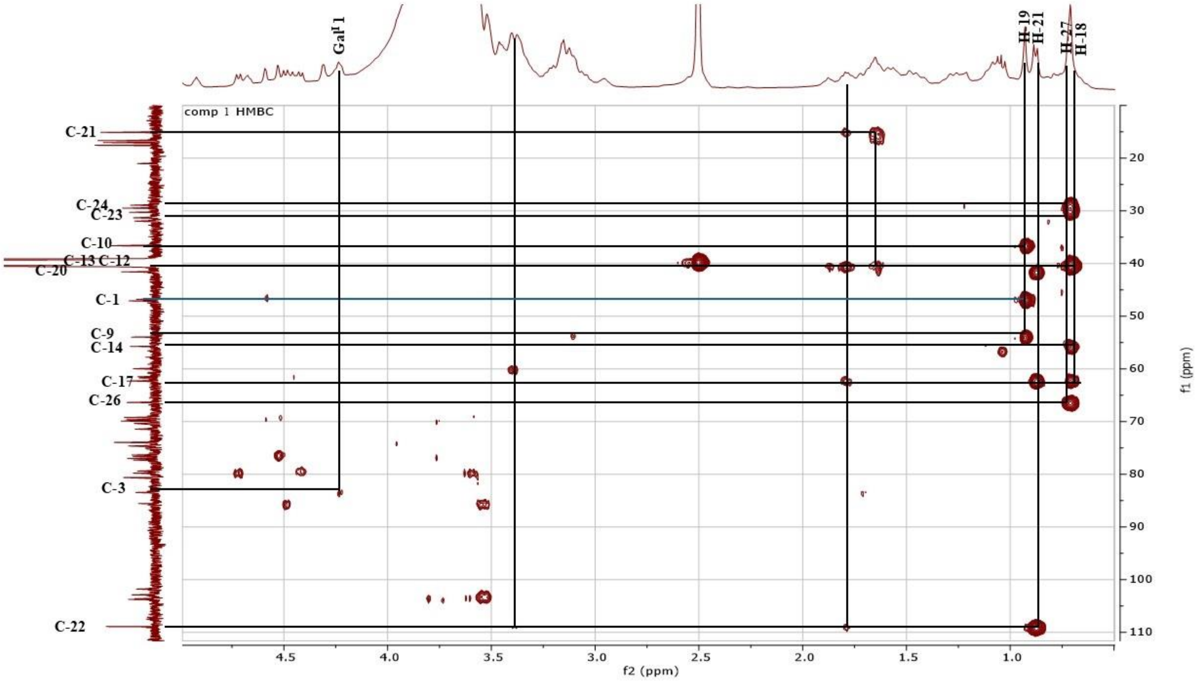

Figure S6. HMBC spectrum of compound 1.

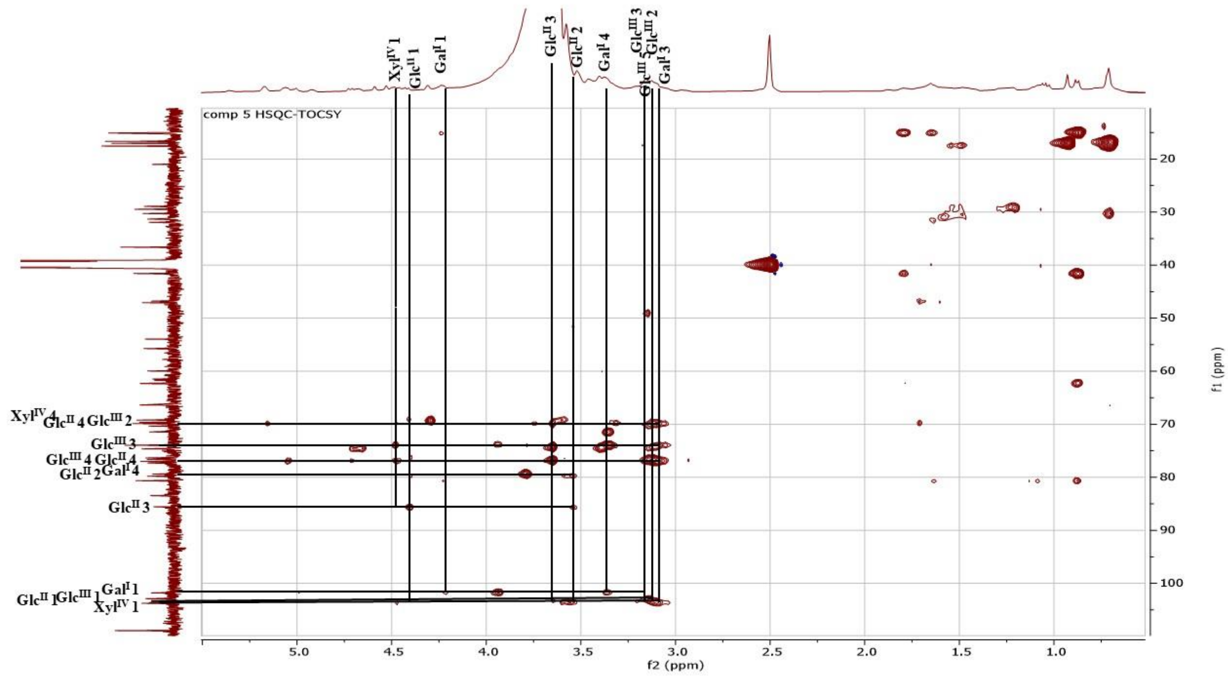

Figure S7. HSQC-TOCSY spectrum of compound 1.

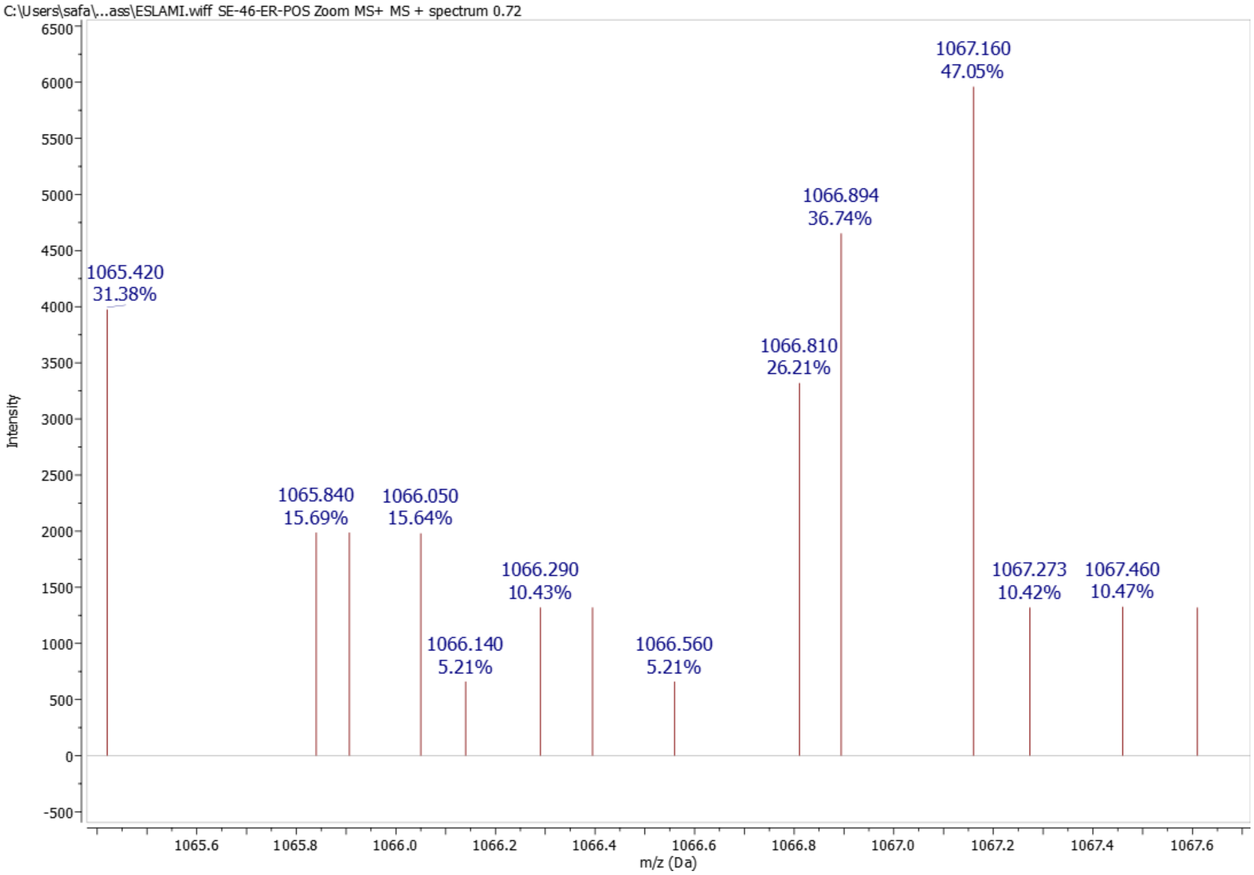

Figure S8. Positive ESI Mass spectrum of compound 1

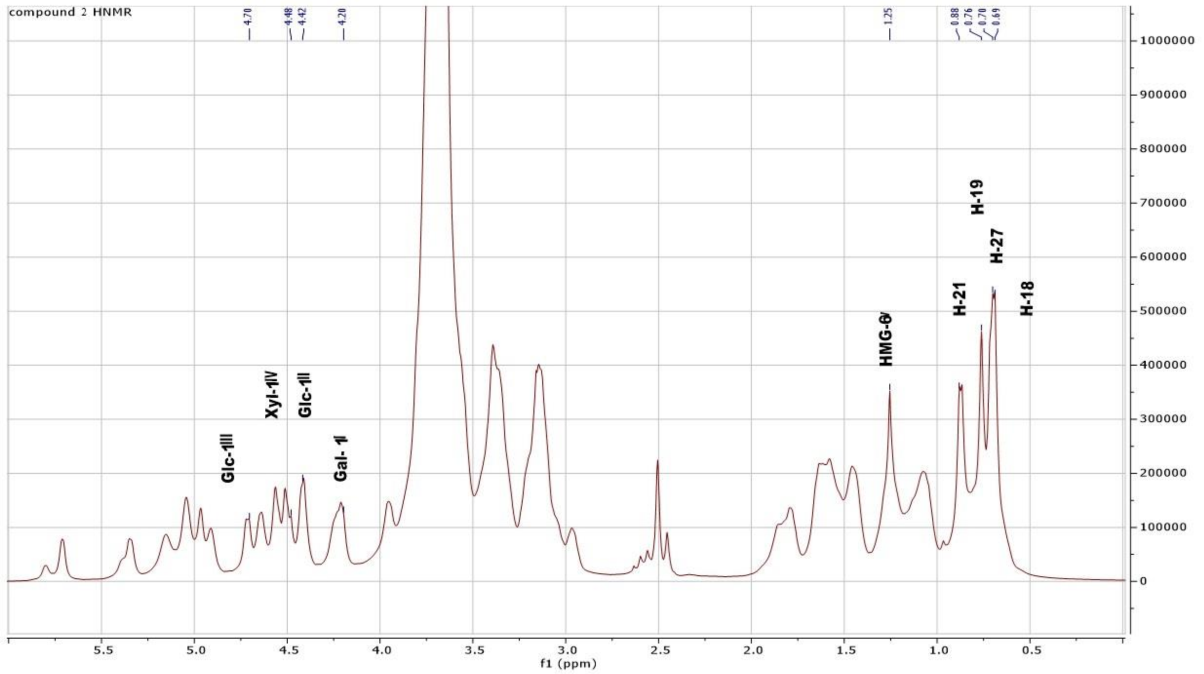

Figure S9. 1H-NMR (400 MHz, DMSO-d6) spectrum of compound 2.

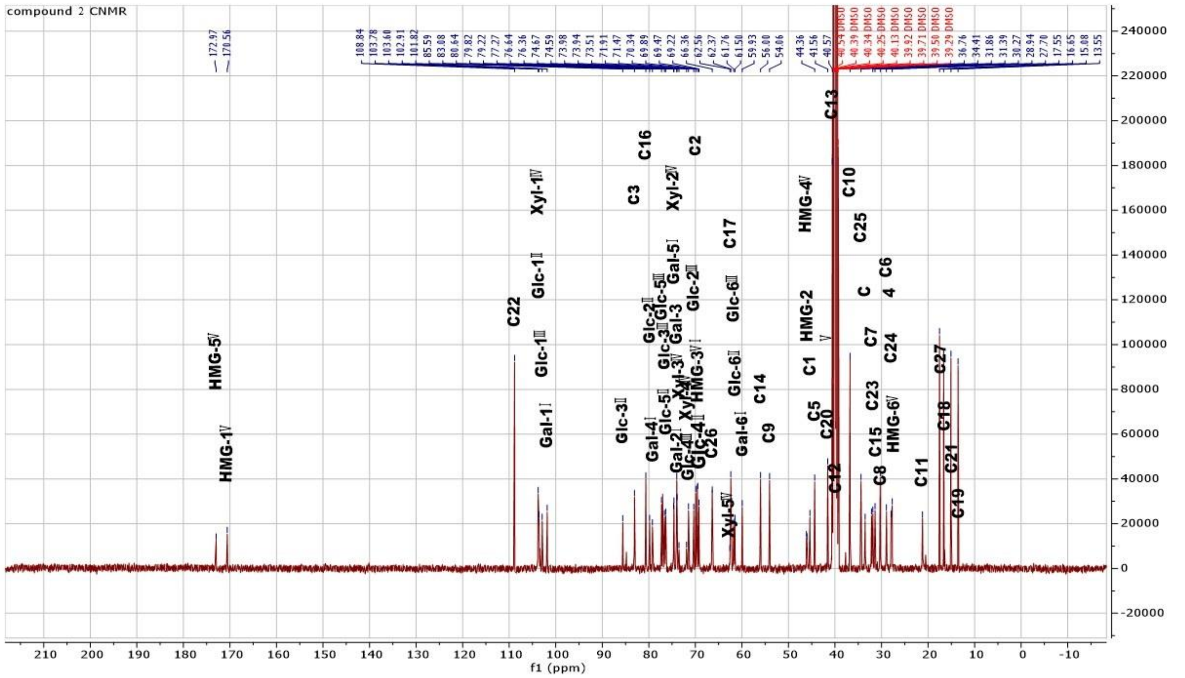

Figure S10. 13C-NMR (100 MHz, DMSO-d6) spectrum of compound 2.

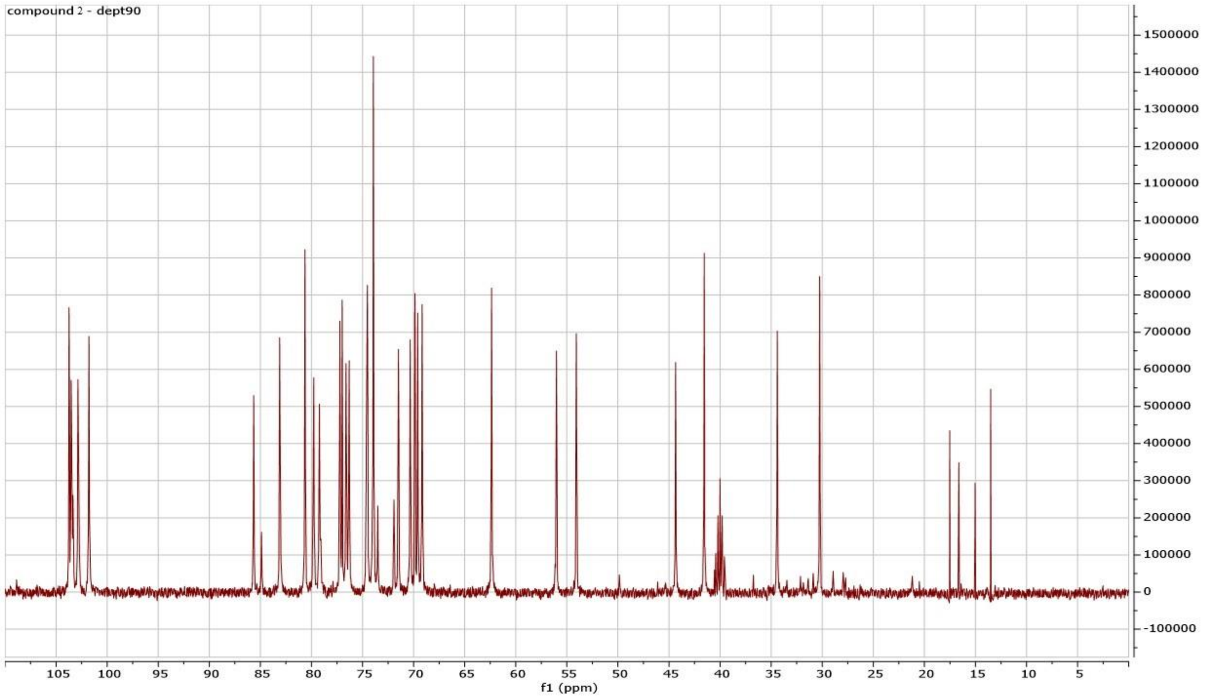

Figure S11. DEPT-90 spectrum of compound 2.

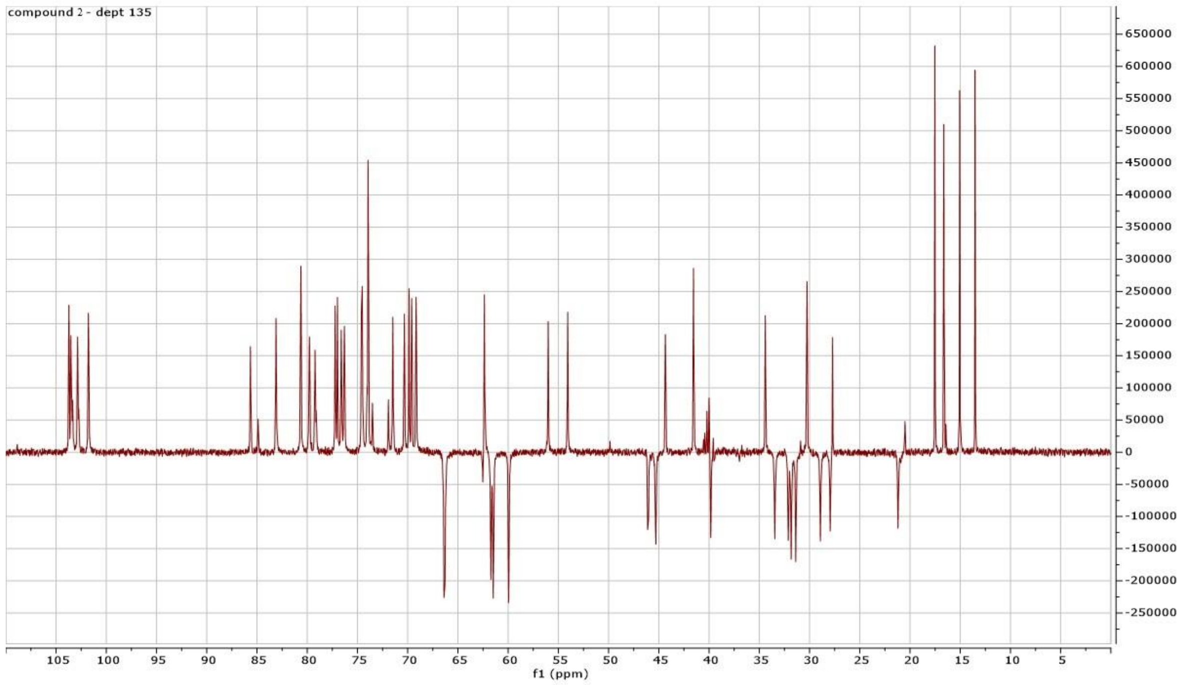

Figure S12. DEPT-135 spectrum of compound 2.

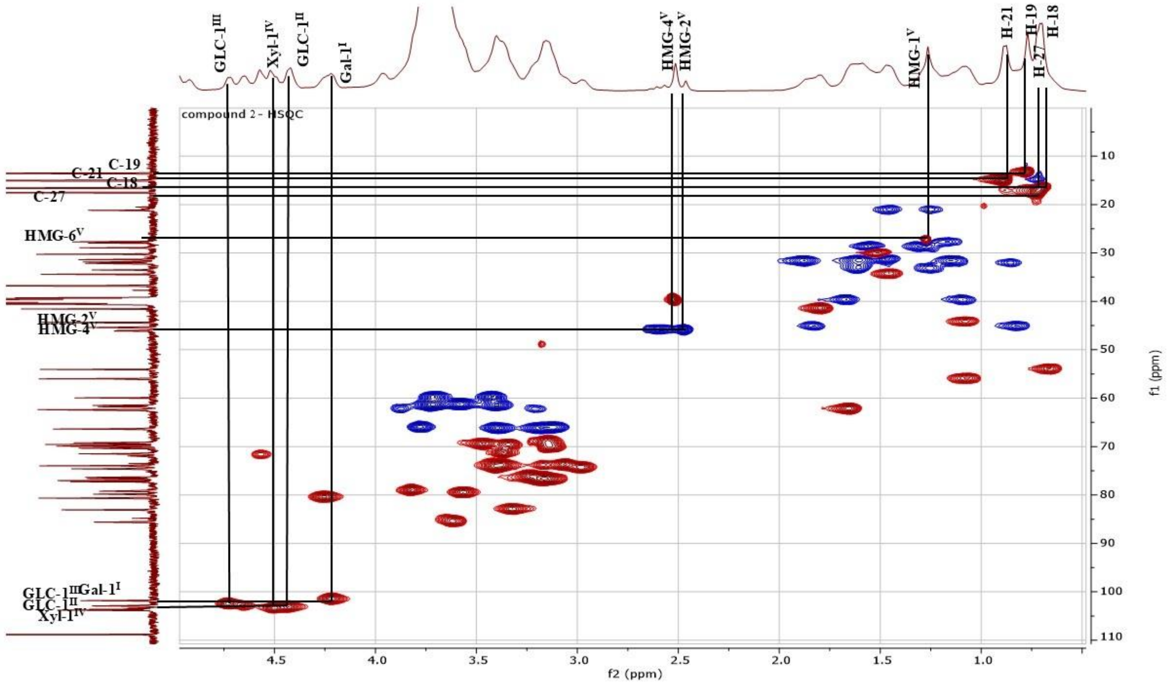

Figure S13. HSQC spectrum of compound 2.

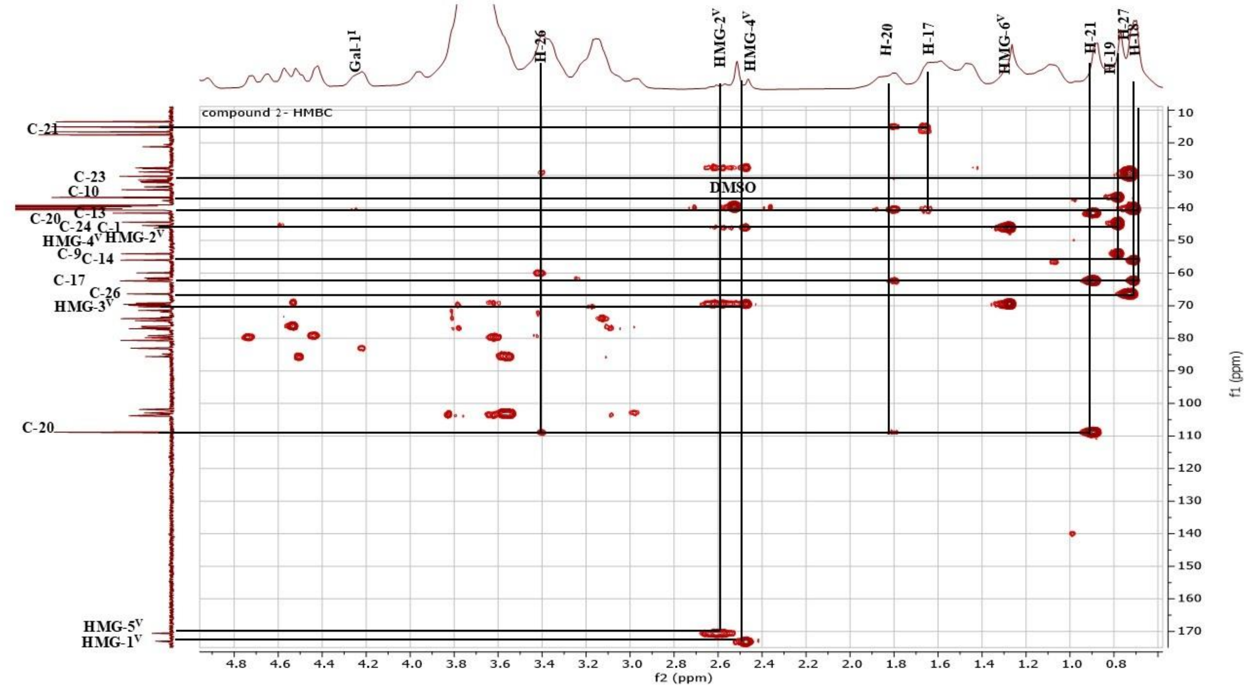

Figure S14. HMBC spectrum of compound 2.

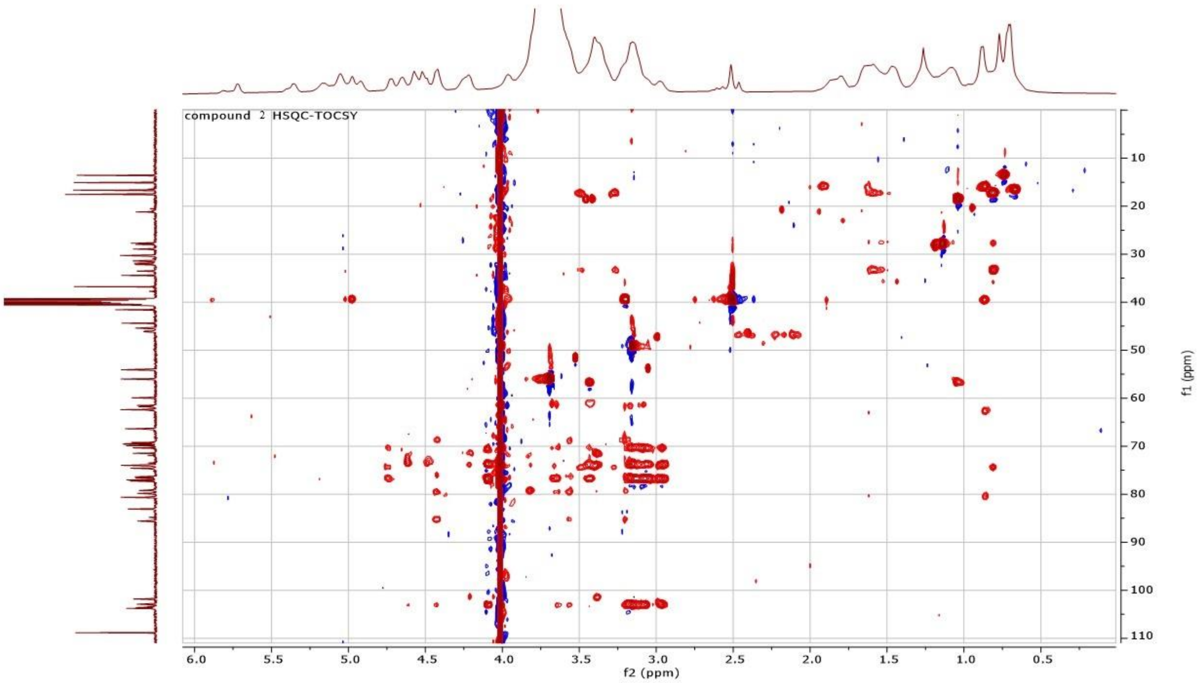

Figure S15. HSQC-TOCSY spectrum of compound 2.

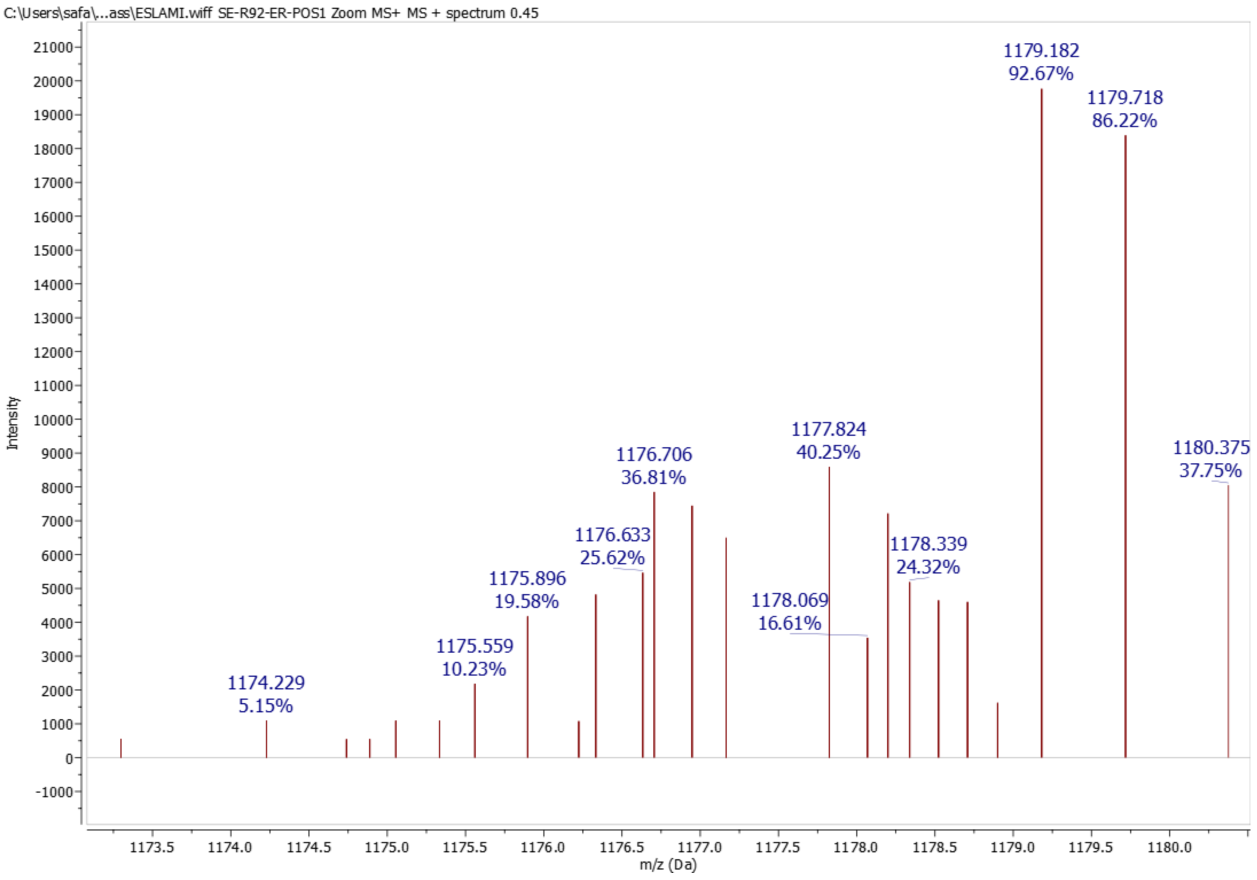

Figure S16. Positive ESI Mass spectrum of compound 2

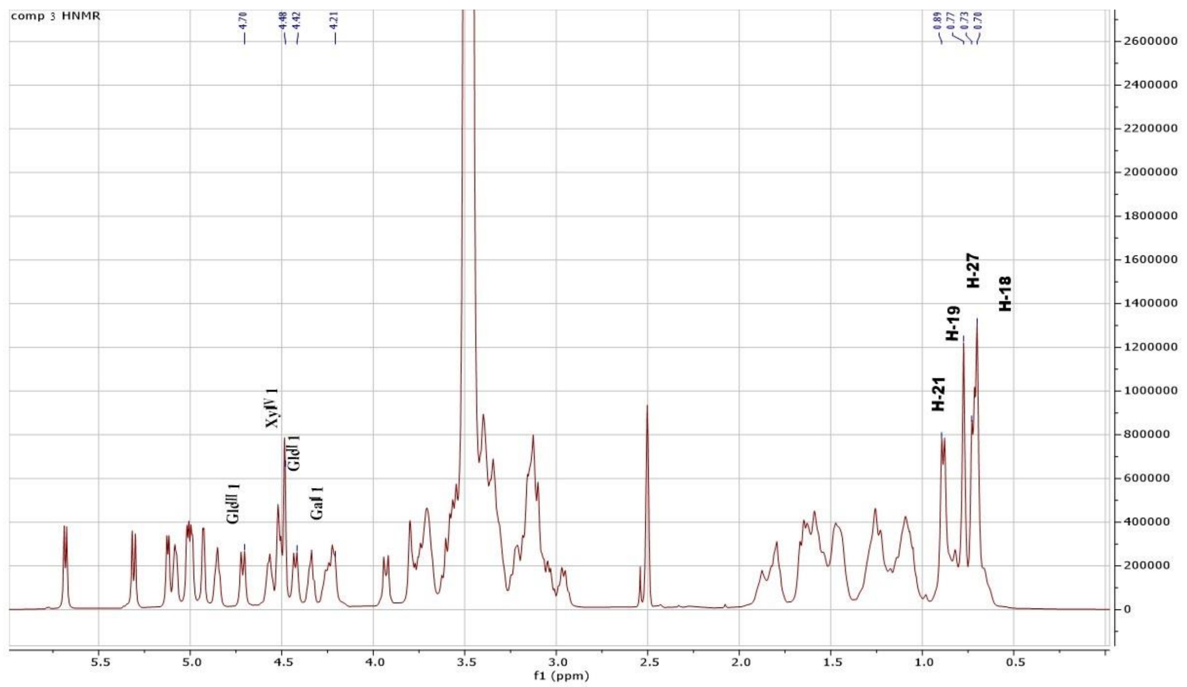

Figure S17. 1H NMR (400 MHz, DMSO-d6) spectrum of compound 3.

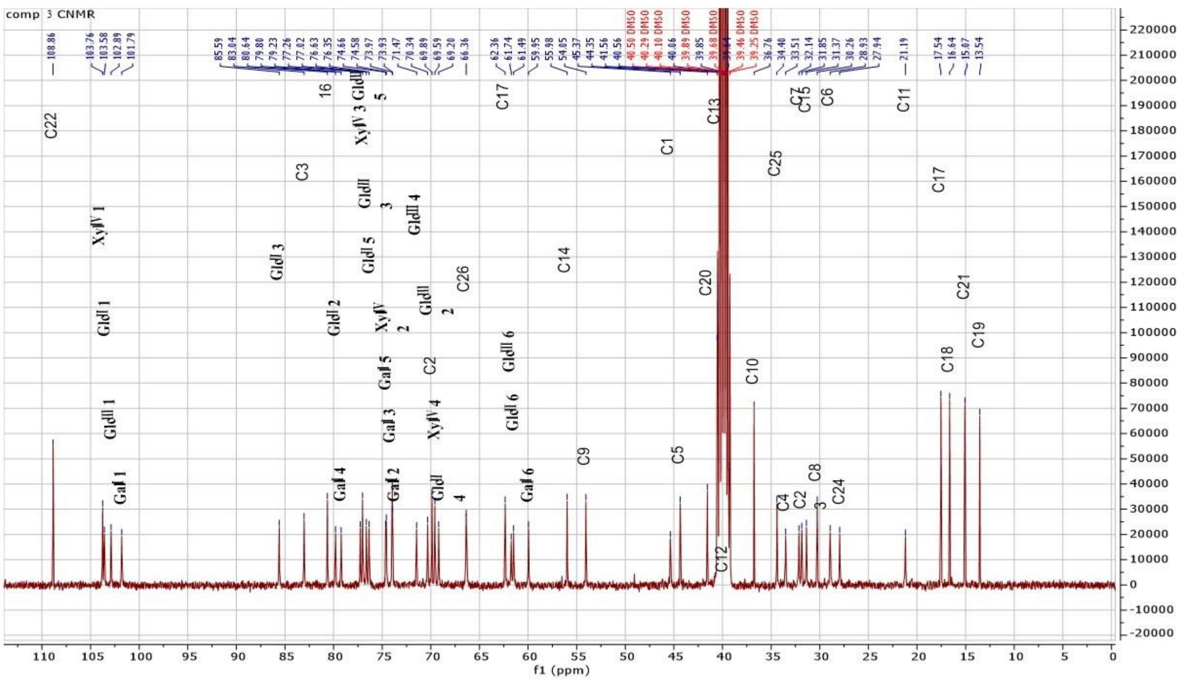

Figure S18. 13C NMR (100 MHz, DMSO-d6) spectrum of compound 3.

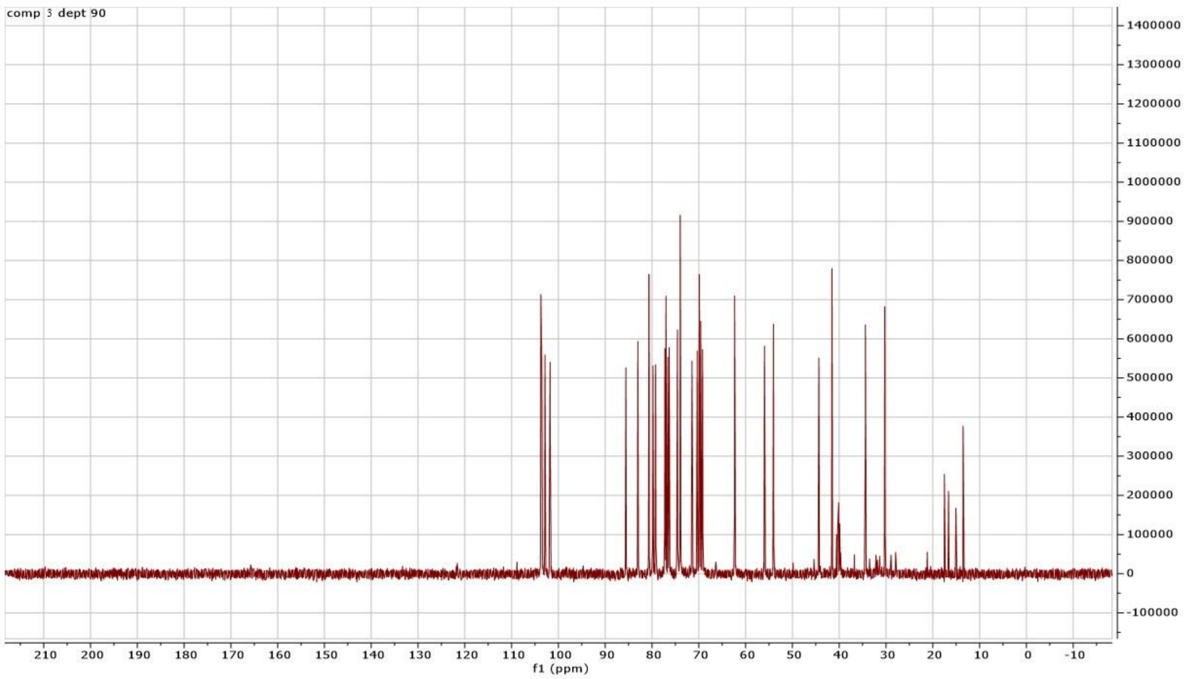

Figure S19. DEPT-90 spectrum of compound 3.

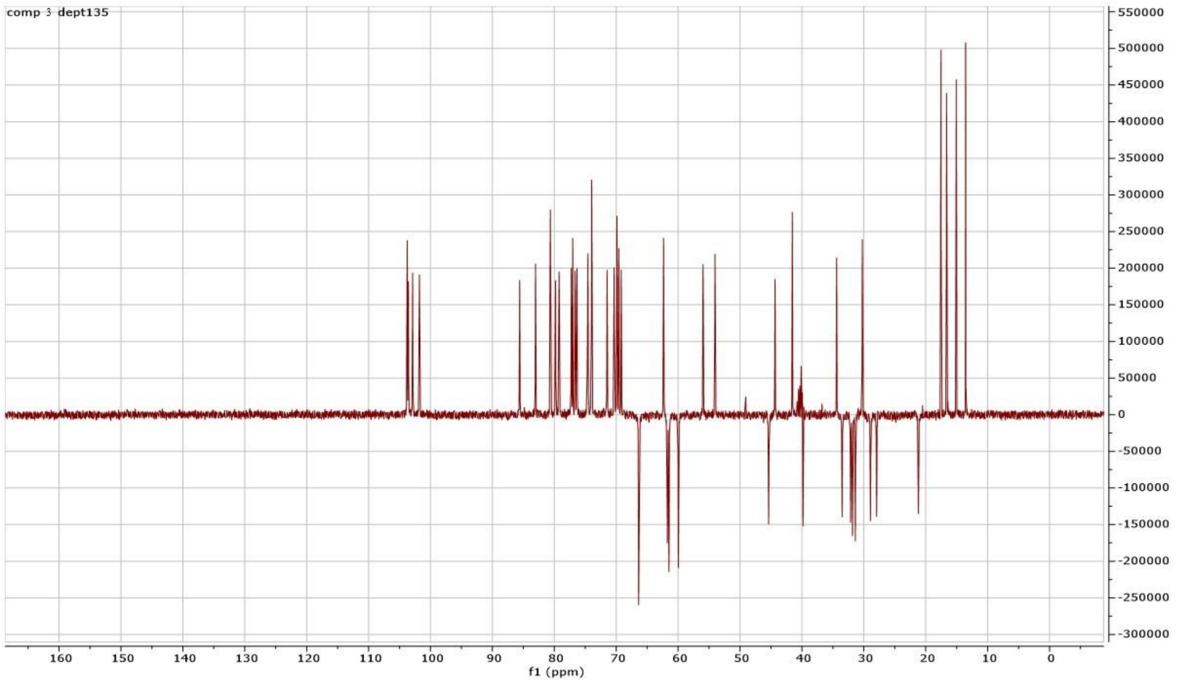

Figure S20. DEPT-135 spectrum of compound 3.

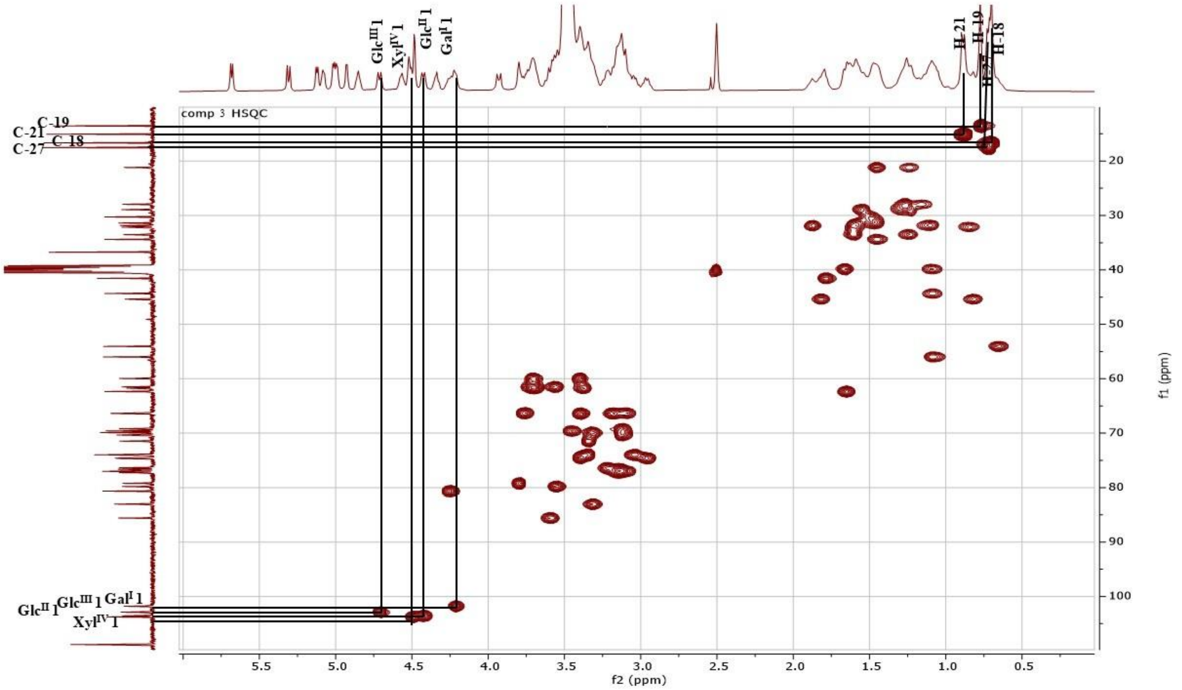

Figure S21. HSQC spectrum of compound 3.

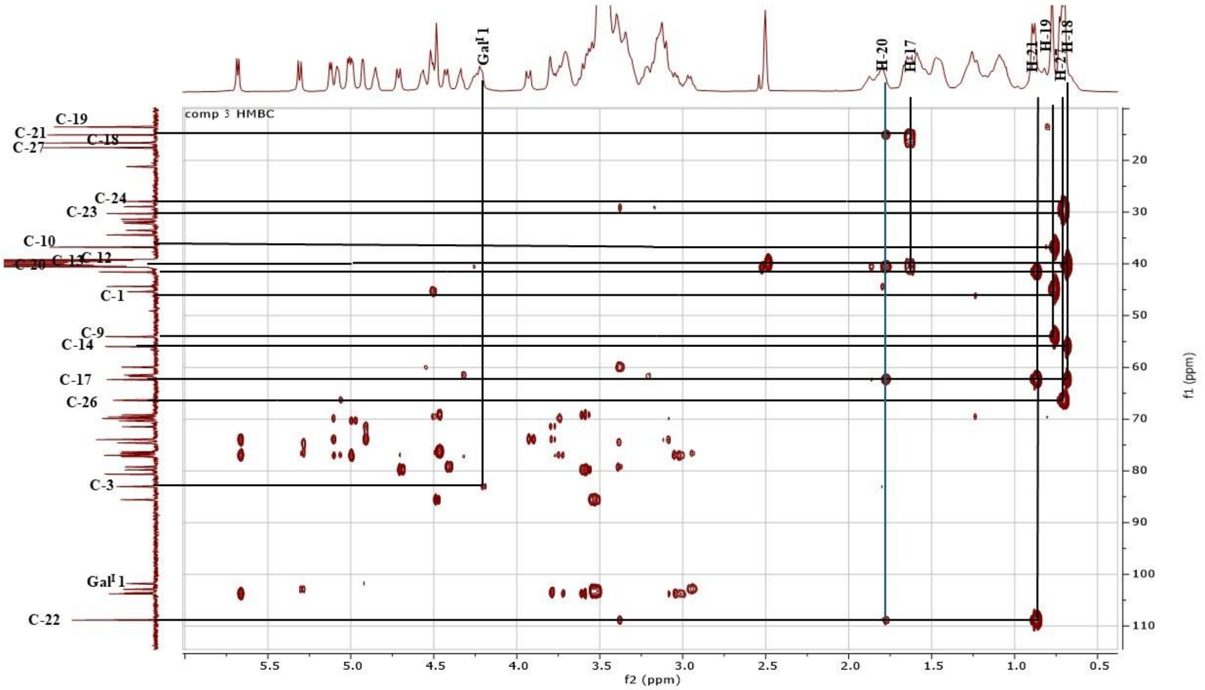

Figure S22. HMBC spectrum of compound 3.

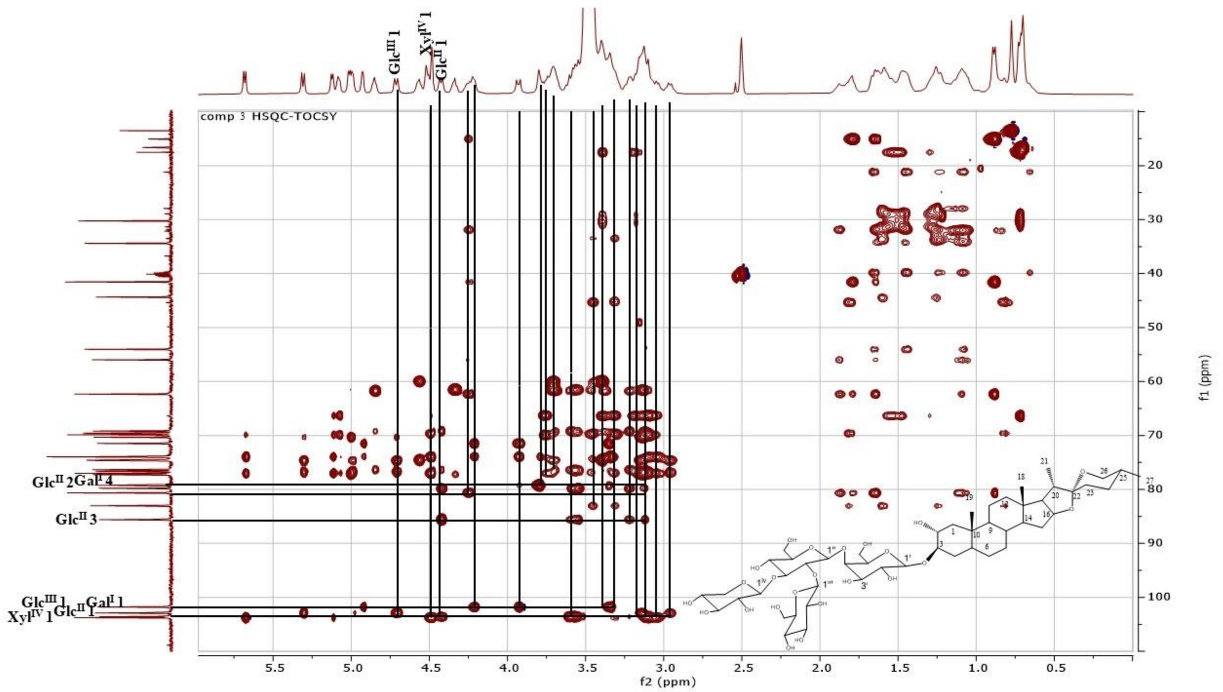

Figure S23. HSQC-TOCSY spectrum of compound 3.

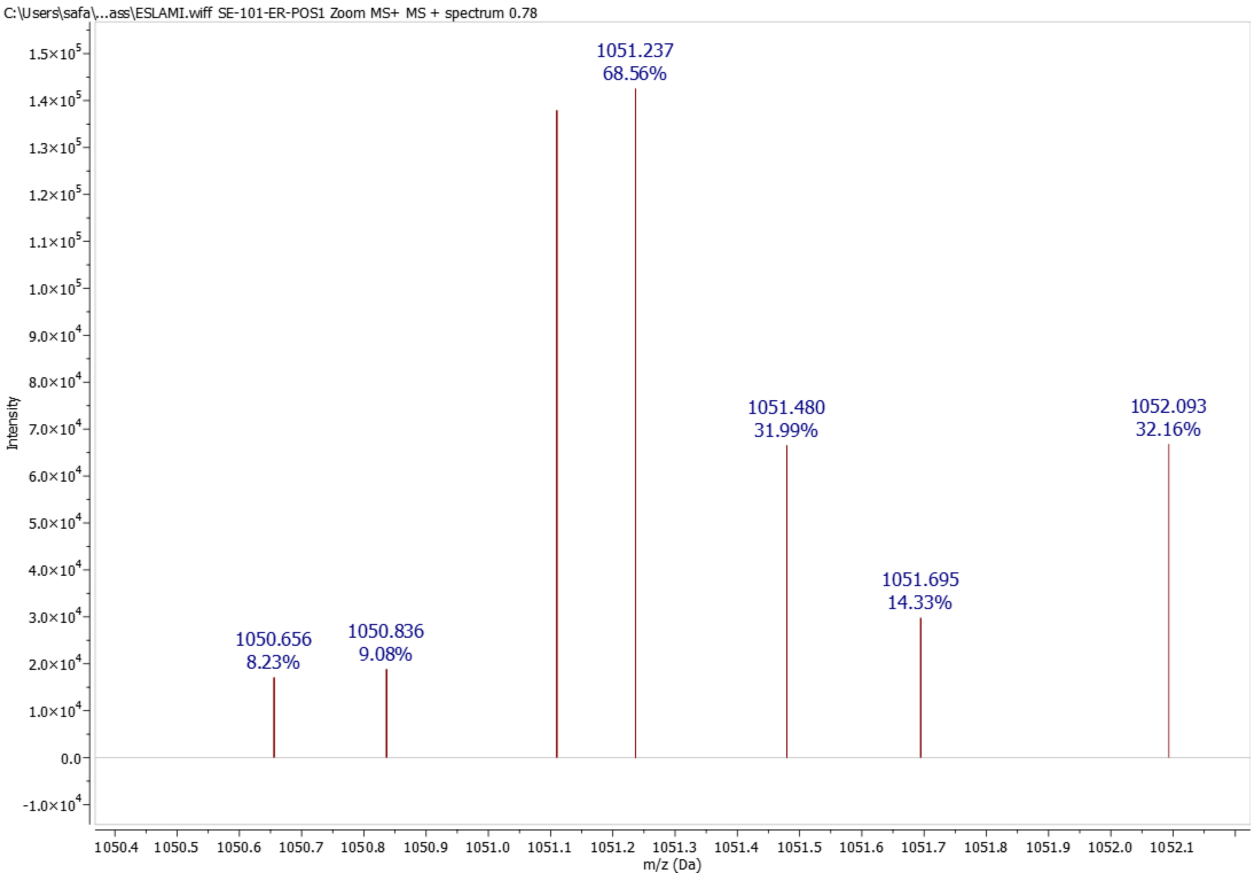

Figure S24. Positive ESI Mass spectrum of compound 3

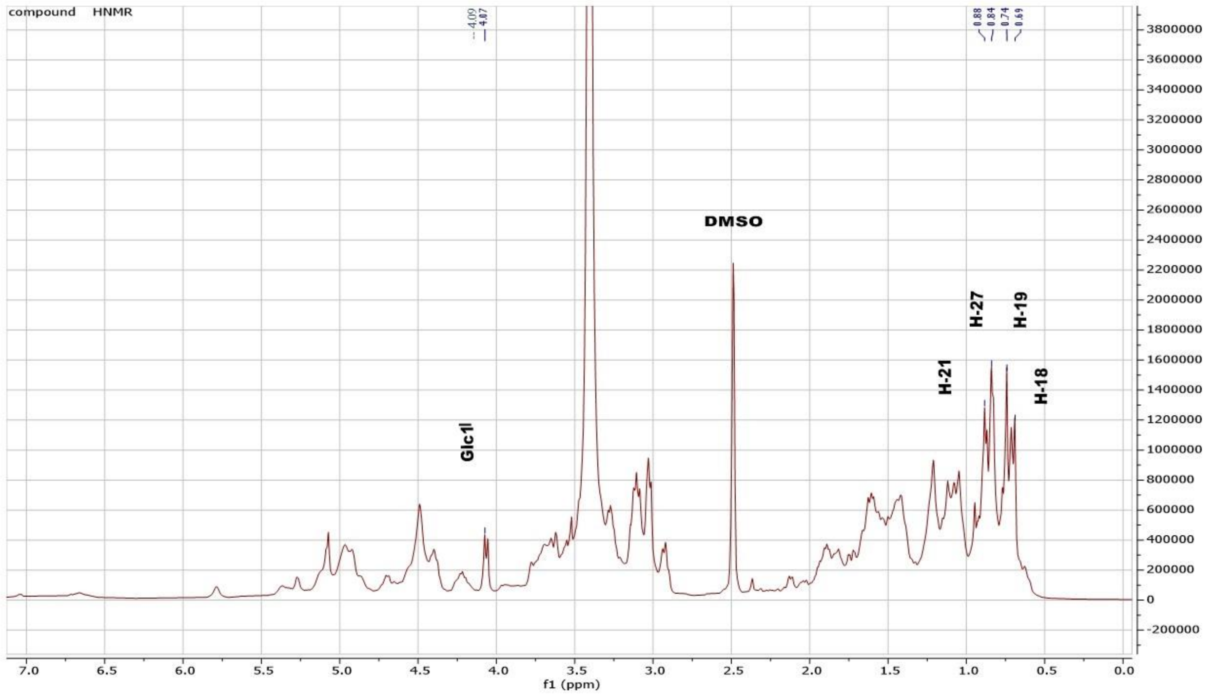

Figure S25. 1H NMR (400 MHz, DMSO-d6) spectrum of compound 4.

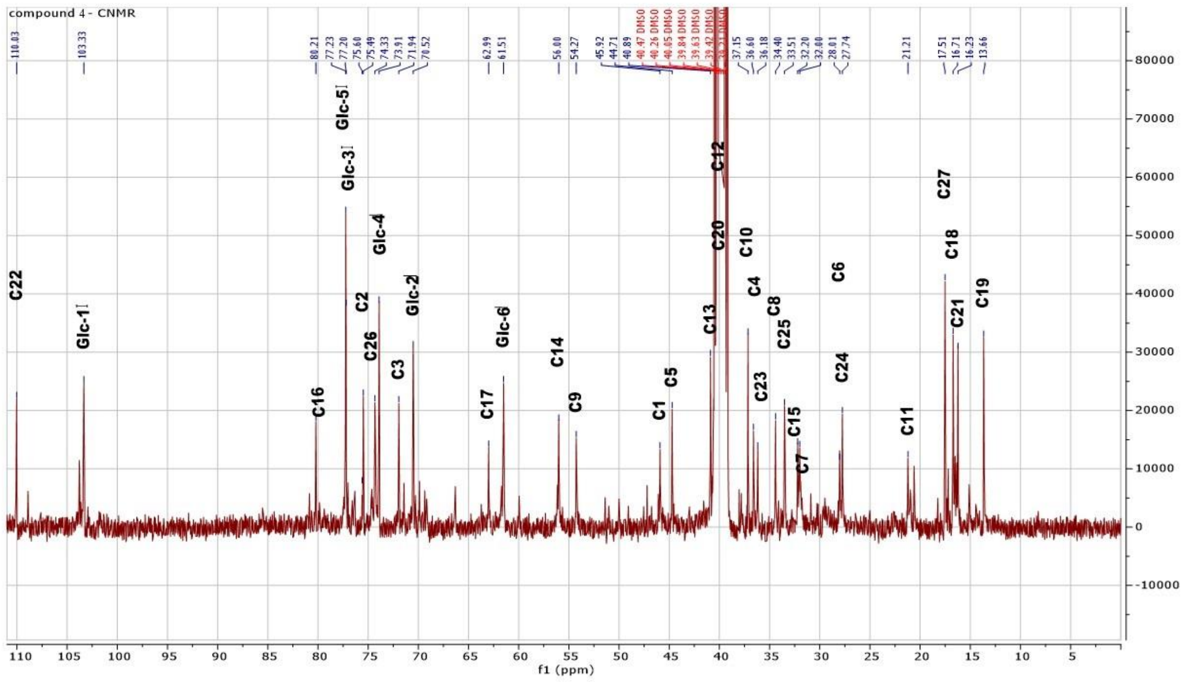

Figure S26. 13C NMR (100 MHz, DMSO-d6) spectrum of compound 4.

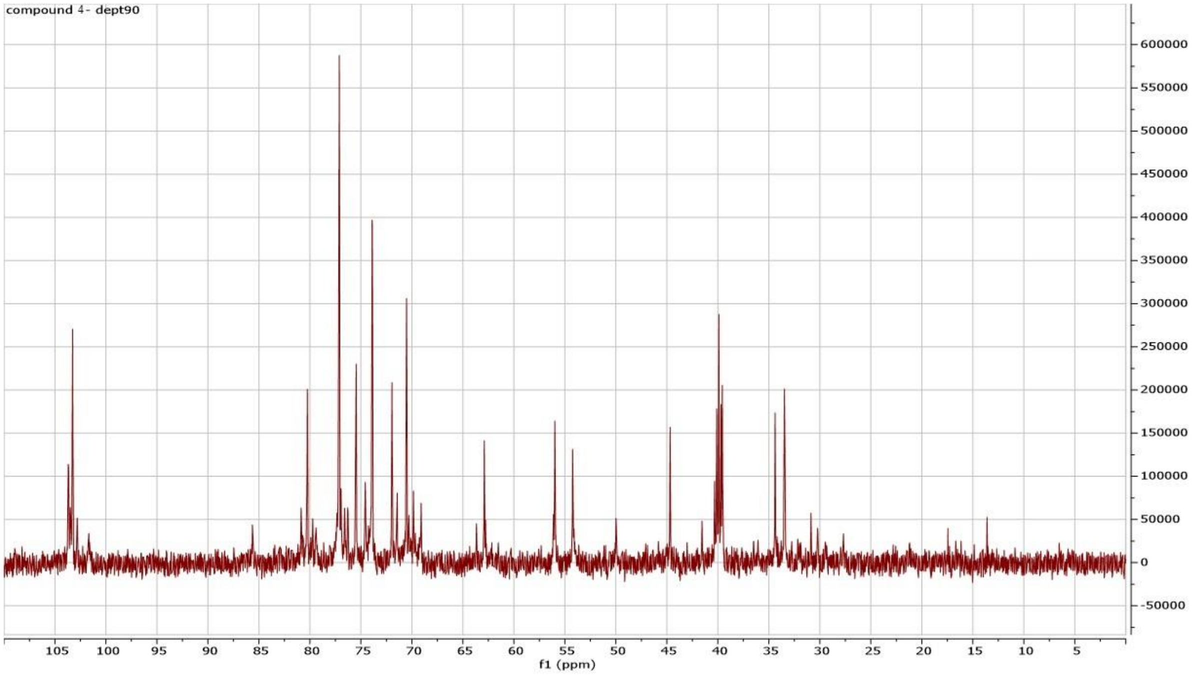

Figure S27. DEPT-90 spectrum of compound 4.

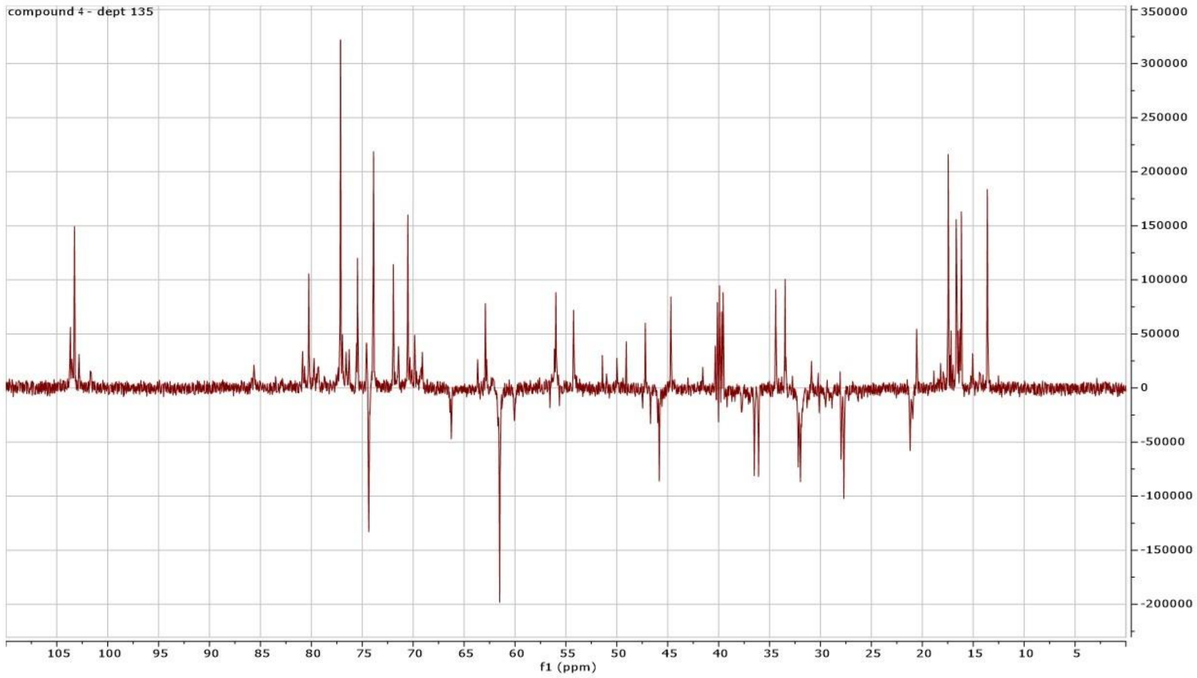

Figure S28. DEPT-135 spectrum of compound 4.

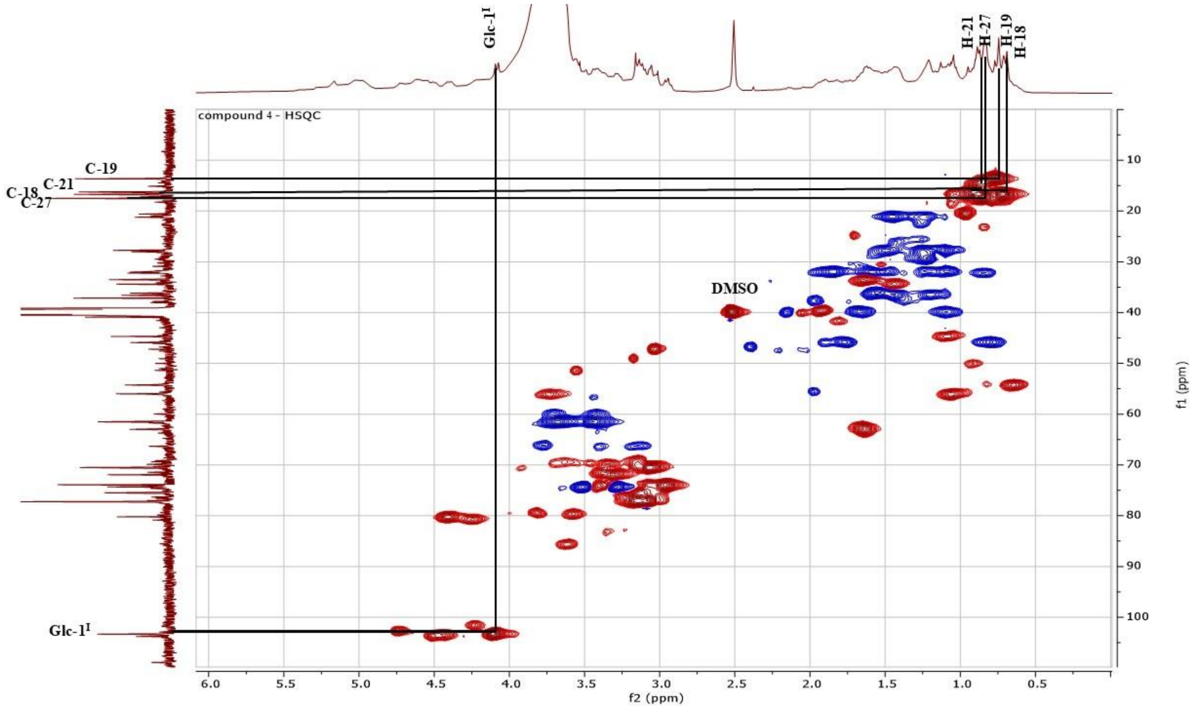

Figure S29. HSQC spectrum of compound 4.

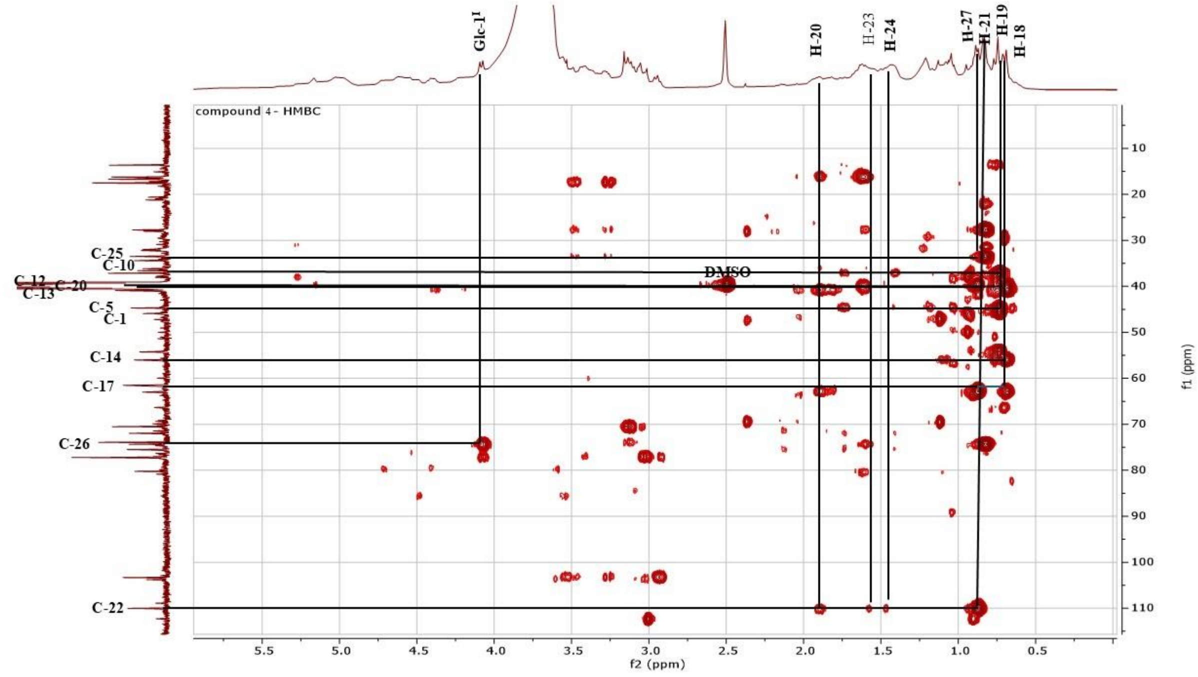

Figure S30. HMBC spectrum of compound 4.

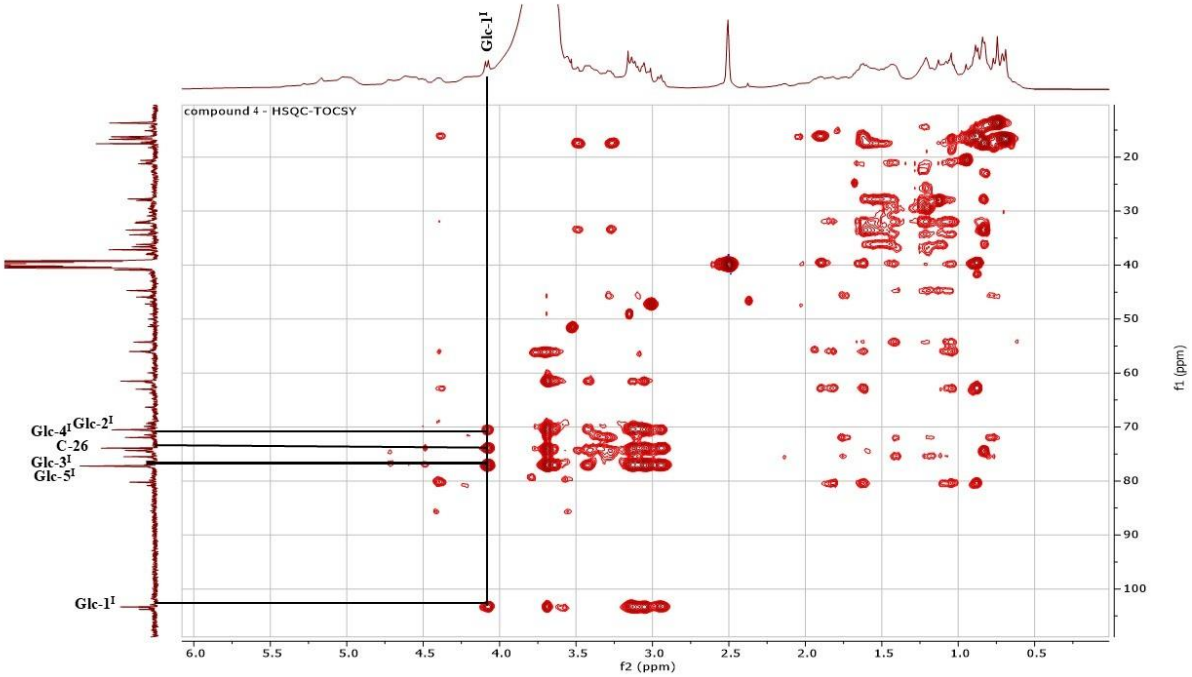

Figure S31. HSQC-TOCSY spectrum of compound 4.

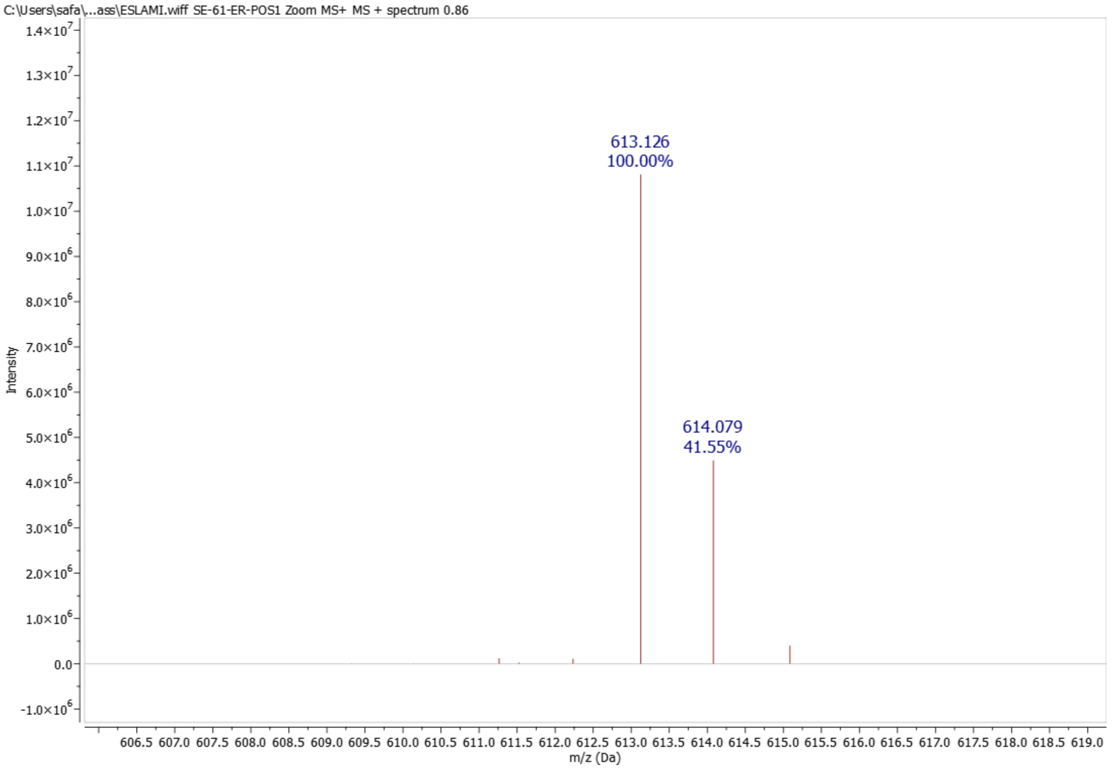

Figure S32. Positive ESI Mass spectrum of compound 4

Table S1. The list of primers sequences for real-time PCR

Primer	Sequence (5'-3')	Product (bp)	References
bla_{KPC}	F: CAACCTCGTCGCGGAACCAT	236	(1)
	R: ACCACGGAACCAGCGCATTT		
*opr*L	F: AACAGCGGTGCCGTTGAC	87	(2)
	R: GTCGGAGCTGTCGTACTCGAA		
*ade*B	F: AACGGACGACCATCTTTGAG	84	(3)
	R: CAGTTGTTCCATTTCACGCA		
*acr*A	F: CTCTCAGGCAGCTTAGCCCTAA	107	(4)
	R: TGCAGAGGTTCAGTTTTGACTGTT		
16SrRNA	F: ATGTTGGGTTAAGTCCCG	256	(5)
	R: CTAGCGATTCCRRCTTCA		

References:

1. Singh K, Mangold KA, Wyant K, Schora DM, Voss B, Kaul KL, Hayden MK, Chundi V, Peterson LR. Rectal screening for *Klebsiella pneumoniae carbapenemases*: comparison of real-time PCR and culture using two selective screening agar plates. J Clin Microbiol. 2012;50(8):2596-600.

2. Joly B, Pierre M, Auvin S, Colin F, Gottrand F, Guery B, Husson MO. Relative expression of Pseudomonas aeruginosa virulence genes analyzed by a real time RT-PCR method during lung infection in rats. FEMS Microbiol Lett. 2005;243(1):271-8.

3. Rafiei E, Shahini Shams Abadi M, Zamanzad B, Gholipour A. The frequency of efflux pump genes expression in *Acinetobacter baumannii* isolates from pulmonary secretions. AMB Express. 2022;12(1):103.

4. Swick MC, Morgan-Linnell SK, Carlson KM, Zechiedrich L. Expression of multidrug efflux pump genes *acrAB-tolC*, *mdfA*, and *norE* in *Escherichia coli* clinical isolates as a function of fluoroquinolone and multidrug resistance. Antimicrob Agents Chemother. 2011;55(2):921-4.

5. Mirzaei A, Nasr Esfahani B, Ghanadian M, Moghim S. *Alhagi maurorum* extract modulates quorum sensing genes and biofilm formation in *Proteus mirabilis*. Sci Rep. 2022;12(1):13992.
